# CRISPR/Cas9-mediated genetic correction reverses spinocerebellar ataxia 3 disease-associated phenotypes in differentiated cerebellar neurons

**DOI:** 10.1093/lifemedi/lnac020

**Published:** 2022-06-29

**Authors:** Guoxu Song, Yuying Ma, Xing Gao, Xuewen Zhang, Fei Zhang, Chunhong Tian, Jiajia Hou, Zheng Liu, Zixin Zhao, Yong Tian

**Affiliations:** Laboratory of RNA Biology, Institute of Biophysics, Chinese Academy of Sciences, Beijing 100101, China; University of Chinese Academy of Sciences, Beijing 100049, China; Laboratory of RNA Biology, Institute of Biophysics, Chinese Academy of Sciences, Beijing 100101, China; University of Chinese Academy of Sciences, Beijing 100049, China; Laboratory of RNA Biology, Institute of Biophysics, Chinese Academy of Sciences, Beijing 100101, China; Laboratory of RNA Biology, Institute of Biophysics, Chinese Academy of Sciences, Beijing 100101, China; Laboratory of RNA Biology, Institute of Biophysics, Chinese Academy of Sciences, Beijing 100101, China; University of Chinese Academy of Sciences, Beijing 100049, China; Laboratory of RNA Biology, Institute of Biophysics, Chinese Academy of Sciences, Beijing 100101, China; University of Chinese Academy of Sciences, Beijing 100049, China; Laboratory of RNA Biology, Institute of Biophysics, Chinese Academy of Sciences, Beijing 100101, China; Laboratory of RNA Biology, Institute of Biophysics, Chinese Academy of Sciences, Beijing 100101, China; Laboratory of RNA Biology, Institute of Biophysics, Chinese Academy of Sciences, Beijing 100101, China; Laboratory of RNA Biology, Institute of Biophysics, Chinese Academy of Sciences, Beijing 100101, China; University of Chinese Academy of Sciences, Beijing 100049, China

**Keywords:** genetic correction, spinocerebellar ataxia 3, iPSC, CRISPR/Cas9, cell differentiation

## Abstract

The neurodegenerative disease spinocerebellar ataxia type 3 (SCA3; also called Machado-Joseph disease, MJD) is a trinucleotide repeat disorder caused by expansion of the CAG repeats in the *ATXN3* gene. Here, we applied a CRISPR/Cas9-mediated approach using homologous recombination to achieve a one-step genetic correction in SCA3-specific induced pluripotent stem cells (iPSCs). The genetic correction reversed disease-associated phenotypes during cerebellar region-specific differentiation. In addition, we observed spontaneous ataxin-3 aggregates specifically in mature cerebellar neurons differentiated from SCA3 iPSCs rather than in SCA3 pan-neurons, SCA3 iPSCs or neural stem cells, suggesting that SCA3 iPSC-derived disease-specific and region-specific cerebellar neurons can provide unique cellular models for studying SCA3 pathogenesis *in vitro*. Importantly, the genetically corrected cerebellar neurons did not display typical SCA3 aggregates, suggesting that genetic correction can subsequently reverse SCA3 disease progression. Our strategy can be applied to other trinucleotide repeat disorders to facilitate disease modeling, mechanistic studies and drug discovery.

## Introduction

Spinocerebellar ataxia type 3 (SCA3) or Machado-Joseph disease (MJD) is an autosomal dominant inherited neurodegenerative disease that belongs to the family of trinucleotide repeat disorders. It is the most prevalent spinocerebellar ataxia (SCA) in the world and the second most common polyglutamine (polyQ) disease after Huntington’s disease [1]. Previous studies have shown that the cerebellum was one of the most severely affected brain regions in SCA3 patients [2]. The major clinical symptoms of SCA3 disease include progressive ataxia, balance and gait discoordination, dysarthria and dysphagia [3]. SCA3 is caused by an expansion of CAG repeats, which encodes an abnormally long polyQ tract in the *ATXN3* gene on chromosome 14 [4]. Healthy individuals contain approximately 13–43 CAG repeats, whereas SCA3 patients typically possess 52–86 repeats within the *ATNX3* locus [1, 5]. Ataxin-3 protein (ATXN3) expressed by *ATXN3* gene is a deubiqutinating enzyme, which manifests a ubiquitous expression among different tissues and cell types [6]. The expansion of the CAG repeats is thought to lead to the formation of intracellular ataxin-3 (ATXN3) aggregates, which is the neuropathological hallmark of SCA3 disease [1, 6–8]. However, until now, the underlying pathogenic mechanism has remained elusive.

Genetic manipulation in a variety of cells and organisms has proven to be a promising strategy for deciphering gene function and disease mechanisms. Genome editing techniques have emerged as a powerful tool to develop desired gain-of-function or ­loss-of-function alleles in a simple and efficient manner. Previous studies have demonstrated that the Clustered Regularly Interspaced Short Palindromic Repeats (CRISPR)/CRISPR-associated (Cas) system enabled genome modification in different models [9–11]. In comparison to other genome editing tools such as transcription ­activator-like endonucleases (TALENs) or zinc-finger nucleases (ZFNs), the CRISPR/Cas9 system utilizes an RNA-guided endonuclease to cleave DNA sequences upstream of a “protospacer adjacent motif” (PAM) [12, 13]. The subsequently induced double-strand breaks (DSBs) are repaired either by nonhomologous end-joining (NHEJ), which introduce indel mutations causing frameshifts and premature stop codons, or by homology-directed repair (HDR) through donor templates for more precise genome modification [14, 15].

Human induced pluripotent stem cells (iPSCs) derived from disease patients have become a powerful source to allow for gene correction and model neurological disease *in vitro*. The CRISPR/Cas9 system has been successfully utilized to perform genetic correction in mouse models as well as human disease cell lines [16–18]. Genetically corrected or engineered iPSCs provide a powerful tool to study disease mechanisms when differentiated into various cell lineages, especially disease-specific cell types since genetically corrected or engineered iPSCs share an isogenic background [19]. In this study, we established SCA3 ­disease-specific iPSCs from urine-derived cells of the SCA3 patients and developed efficient methods for one-step genetic correction in SCA3 iPSCs. Furthermore, we differentiated these iPSCs to cerebellar neural stem cells (NSCs) and cerebellar neurons, demonstrating that these region-specific and ­disease-specific differentiated cells can be used as unique cellular models for studying SCA3 pathogenesis *in vitro*.

## Results

### Derivation of SCA3 and WT iPSCs from urinary epithelial cells

**Figure 1.**
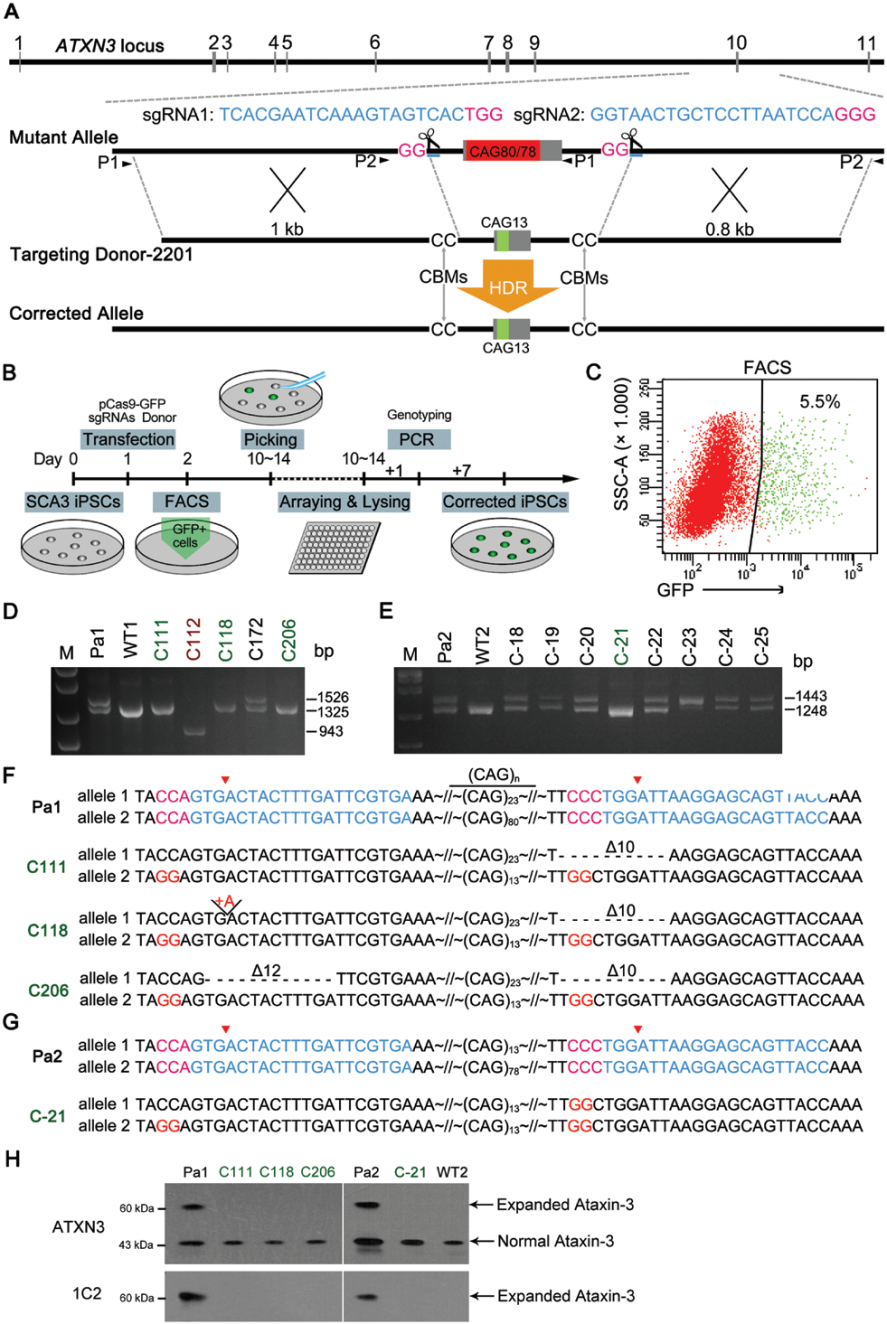
CRISPR/Cas9-mediated genetic correction of SCA3-specific iPSCs using an “intron-based strategy”. (A) A schematic view of a genetic correction strategy of the mutant *ATXN3* allele in SCA3 iPSCs. Gray boxes represent exons at the *ATXN3* locus. The mutant *ATXN3* allele was corrected using CRISPR/Cas9-mediated HDR by adjusting the pathological 80 or 78 CAG repeats (red box) to a normal 13 CAG repeats (green box). The Cas9 protein-blocking mutations (CBMs) (GG to CC) at the PAM region (magenta) were introduced in the donor arms, preventing undesired editing of the target site (blue). P1 and P2 represent PCR primers for amplification. (B) Procedure of targeted correction of SCA3 iPSCs. FACS, fluorescence-activated cell sorting. (C) The ratio of GFP-positive iPS single cells by FACS after 1 day of transfection with the pCas9-GFP plasmid, sgRNA expression vectors and donor. (D and E) PCR-based screen of genetically corrected iPSC clones after gene targeting in Pa1 and Pa2-SCA3 iPSCs. The corrected clones and represented are indicated in green. An uncorrected clone with a large-scale deletion is indicated in red. M, marker. (F and G) Sequencing results of the genetically corrected clones (C111, C118, C206, C-21) near the region of CAG repeats, including the sgRNA target sites (blue) and PAM (magenta). (CAG)n, CAG repeat number; red triangles, cleavage sites; Δ10, delete 10 base pairs; +A, insert an adenine. (H) Western blot analysis indicated that C111, C118, C206, C-21 and WT iPSCs only have a normally sized ataxin-3 protein detected by the ATXN3-specific and polyQ-specific antibody 1C2, when compared to Pa1 and Pa2-SCA3 iPSCs.

First, we collected urinary samples from two severe SCA3 patients (termed Pa1 and Pa2) and two healthy donors (termed WT1 and WT2) and then cultured urinary epithelial cells (Fig. S1A). Subsequently, the urinary epithelial cells were reprogrammed to non-integrating iPSCs by introducing the Yamanaka factors via episomal vectors [20]. These expanded iPS clones exhibited a typical human iPSC morphology and normal karyotype (Figs. S1B and S1D). Immunohistochemistry staining results revealed human pluripotent marker expression, including OCT4, SSEA4, NANOG and SOX2 (Fig. S1C). Expansion of the CAG sequence in exon 10 of the *ATXN3* gene in different cell lines was verified by PCR analysis (Fig. S1F). Furthermore, we confirmed the CAG expansion number in two SCA3 iPSCs by direct sequencing. We found that the mutant *ATXN3* allele contained 80 and 78 CAG repeats in the Pa1-SCA3 and Pa2-SCA3 iPSCs, respectively. And the normal allele contained 23 and 13 CAG repeats in the Pa1-SCA3 and Pa2-SCA3 iPSCs, respectively (Figs. 1F, 1G and S1E). The length of the polyQ repeats was not changed in our experiments for at least 40 passages as iPSCs and subsequent derived neurons (data not shown).

### Genetic correction of CAG repeats in SCA3 iPSCs using an “intron-based strategy”

The *ATXN3* gene, spanning a genomic region of approximately 48 kb, is composed of 11 exons. A CAG repeat expansion (> 52 CAGs) in exon 10, encoding for an expanded polyQ tract, is responsible for SCA3. We searched all of the possible single-guide RNA (sgRNA) locations directly adjacent to the expanded CAG stretch and identified the sgRNA1 target site located in intron 9 and the sgRNA2 target site in intron 10 as suitable for genetic correction (Figs. 1A and S2A). Thus, we applied this “intron-based strategy” for genetic correction in SCA3 iPSCs. To improve HDR efficiency, we established a donor plasmid construct of 2201 bp containing 13 CAG repeats flanked by two homologous arms of approximately 1 kb and 0.8 kb respectively (Fig. 1A). To avoid Cas9 protein cleavage in the donor plasmid and prevent continuous cleavage in sgRNA-binding sites, we artificially introduced two Cas9 protein-blocking mutations (CBMs) in the homologous arms, in which GG was mutated to CC, thus, leading to an unrecognized PAM region (Fig. 1A).

To obtain genetically corrected iPS clones, SCA3 iPSCs were transfected with the pCas9-GFP plasmid, sgRNA expression vectors and donor plasmids (Fig. 1B). One day post transfection, we sorted the cells through fluorescence-activated cell sorting (FACS) and plated GFP-positive cells into a feeder-covered dish supplied with ROCK inhibitor Y-27632. GFP-positive cells were then passed through FACS at a ratio of 5% and up to 17.6% (Figs. 1C and S2C). We further cultured isolated clones for two more weeks before the genomic DNA was extracted for genotyping assay. We screened the genomic DNA from these iPSCs using primer pairs P1 and P2 (Fig. 1A). To exclude false positives, such as plasmid-based amplification, the location from one of the primer pairs was designed to be external to the homology arm (Fig. 1A). The mutant allele displayed 1443-bp and 1526-bp bands using the P1 and P2 primer pair, respectively. But the corrected allele (CAG13) displayed 1248-bp and 1325-bp bands by the P1 and P2 primer pair, respectively (Figs. 1D, 1E and S2B). We also obtained a few uncorrected clones such as clone 112 (C112), which displayed a 943-bp band using the P2 primer pair, perhaps due to a large-scale deletion between the sgRNAs (Fig. 1D). Meanwhile we also preliminarily excluded random donor DNA integration in the iPS genome by PCR using a primer pair D2201-T amplifying donor DNA vector, which yields a 1503-bp band (Fig. S2E). Subsequently, all corrected candidates were further analyzed and verified by Sanger sequencing, Western blot analysis and *ATXN3* cDNA sequencing (Figs. 1 and S4).

Using this intron-based targeting strategy, we screened 218 clones for the Pa1-SCA3 iPSCs and 90 clones for the Pa2-SCA3 iPSCs. A total of four clones were verified to be correctly targeted, which the three clones (C111, C118 and C206) were targeted from Pa1-SCA3 iPSCs and one clone (C-21) from Pa2-SCA3 iPSCs (Table 1). Sequencing results revealed that these clones (C111, C118, C206 and C-21) were corrected by adjusting the mutant CAG allele with expanded CAG repeats to 13 CAG repeats (Figs. 1F, 1G). The normal allele of C-21 clone contained the corrected CBM at sgRNA2 target site, while the normal allele of three clones (C111, C118 and C206) with 23 CAG repeats contained indel mutations at sgRNA-binding sites in intron 9 and 10 (Figs. 1F, 1G, S2D and S2F). To further verify the correction of these clones, we performed the *ATXN3* cDNA sequencing (Fig. S4A) and Western blot analysis (Fig. 1H). The results of cDNA sequencing revealed that the correction events indeed happened at the *ATXN3* locus, in which the number of CAG repeats was 13 or 23 within the exon 10 of *ATXN3* allele in these corrected clones (Figs. S4B–E). Additionally, we did not detect any abnormal band or sequence on *ATXN3* cDNA of these corrected clones, indicating that the intronic CBMs or indels did not affect the *ATXN3* mRNA splicing. Western blot analysis using an ataxin-3-specific antibody revealed that the C111, C118, C206 and C-21 clones expressed only the normal size ataxin-3 protein (~43 kDa) from normal *ATXN3* and corrected *ATXN3* (CAG13) allele, indicating that small intronic deletions did not affect ataxin-3 protein expression, while Pa1 and Pa2-SCA3 iPSCs expressed the normal and expanded ataxin-3 protein (~60 kDa). We failed to detect any signal with Western blot using a polyQ-specific antibody 1C2, which recognizes the expanded polyglutamine stretch, in all 4 clones (C111, C118, C206 and C-21) (Fig. 1H).

**Table 1.** Gene correction efficiencies in SCA3 iPSCs

Patient	Strategy	Cells transfected	GFP + cells sorting	Clones screened [a]	Targeted clones [b] (efficiency = b/a × 100%)	Corrected clones [c] (efficiency = c/b × 100%)	HDR efficiency (d*/2b × 100%) (%)
Pa1	Intron	6 × 10^6^	1 × 10^4^	218	49 (22.5%)	3 (6.1%)	6.1
Pa1	Exon	6 × 10^6^	8 × 10^3^	135	34 (25.2%)	1 (2.9%)	7.3
Pa2	Intron	3 × 10^6^	5 × 10^3^	90	26 (28.9%)	1 (3.9%)	6.1
Pa2	Exon	3 × 10^6^	5 × 10^3^	83	23 (27.7%)	0	6.5

d*: The number of corrected alleles with HDR revealed by Sanger sequencing.

### Genetic correction of CAG repeats in SCA3 iPSCs using an “exon-based strategy”

**Figure 2.**
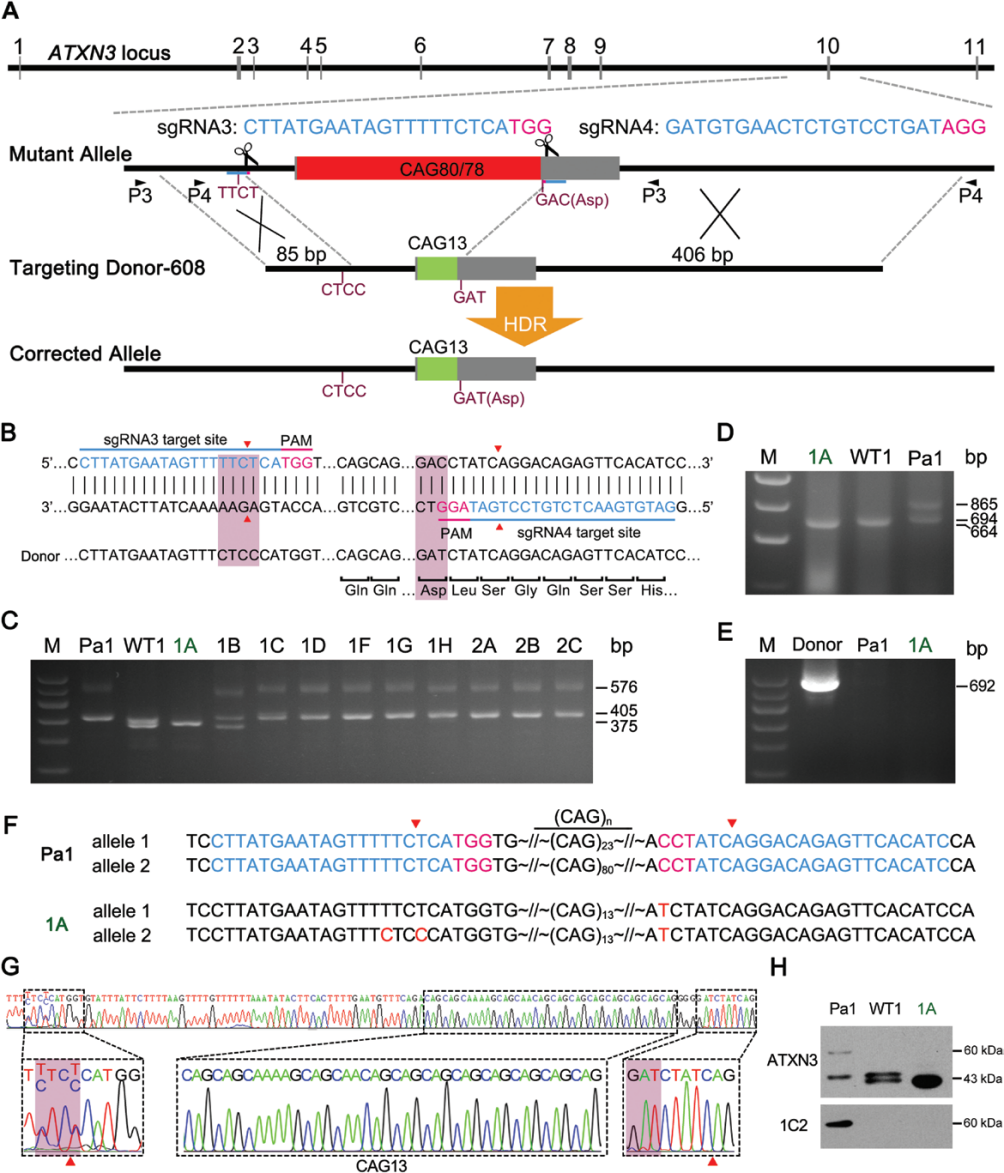
CRISPR/Cas9-mediated genetic correction of SCA3-specific iPSCs using an “exon-based strategy”. (A) The schematic view of the “exon-based strategy”. Donor-608 was used as a template with homologous arms of 81 bp on the left and 406 bp on the right. CBMs (TTCT to CTCC and GAC to GAT) (fuchsia) were introduced to prevent undesired editing of the target site (blue). P3, P4, PCR Primer pair. (B) The overview of CBMs designed in the exon-based strategy, which introduced TTCT to CTCC in the intron and GAC to GAT in the exon, leaving a same-sense mutation but an unrecognized PAM region by Cas9 in the exon. Fuchsia shades represent the sites of the CBMs. Target sites and PAM region are indicated in blue and magenta, respectively. Red triangles, cleavage sites. (C and D) PCR screening of iPS clones with P3 and P4 primer pairs. Corrected clones represented are indicated in green. M, marker. (E) Examination of donor DNA random integration into the chromosomes by PCR using primer pair D608-T. (F) Sequencing analysis of the genetically corrected clone 1A. The CBMs integrated into the genome by HDR are in red. (G) Sequencing results revealed that clone 1A was corrected by adjusting both the mutant CAG allele (CAG80) and WT allele (CAG23) to 13-CAG repeats. Red triangles, cleavage sites; fuchsia shades, the sites of the CBMs. (H) Western blot analysis indicated that clone 1A and WT iPSCs only have a normally sized ataxin-3 protein detected by the ATXN3-specific and polyQ-specific antibody 1C2, when compared to Pa1-SCA3 iPSCs.

To further establish our targeting strategy, we also designed an “exon-based strategy” for genetic correction. We rescreened the sgRNA loci closest to the region of expanded CAG repeats and identified sgRNA3 located in intron 9 and sgRNA4 in exon 10 (Fig. S2A). We utilized a donor template called “Donor-608” with short homology arms for HDR purposes. The Donor-608 construct was 608-bp long, containing 13 CAG repeats flanked by two short homologous arms that were 81-bp and 406-bp on each side (Fig. 2A). We also artificially introduced CBMs within the homology arms by mutating TTCT to CTCC in the left arm and GAC to GAT in the right arm, which generated a same-sense mutation but an unrecognized PAM region in exon 10 (Fig. 2B).

Next, we screened iPS cells using primer pairs P3 and P4 (Fig. 2A). The mutant allele displayed 576-bp and 865-bp bands using the P3 and P4 primer pair, respectively (Figs. 2C, 2D and S3A). But the corrected allele (CAG13) displayed 375-bp and 664-bp bands by the P3 and P4 primer pair, respectively (Figs. 2C, 2D and S3A). Meanwhile we also preliminarily excluded random donor DNA integration in the genome by PCR using a primer pair D608-T amplifying donor DNA vector (Fig. 2E). Subsequently, all corrected candidates were further analyzed and verified by Sanger sequencing, Western blot analysis and *ATXN3* cDNA sequencing.

Using this exon-based targeting strategy, we screened 135 clones for the Pa1-SCA3 iPSCs and 83 clones for the Pa2-SCA3 iPSCs. Only one clone (1A) which was generated from Pa1-SCA3 iPSCs was verified to be correctly targeted (Table 1). Although we detected Pa2-SCA3 targeted clone 6C harboring a corrected allele (CAG13), the normal allele of clone 6C contained an adenine insertion on the sgRNA4 target site in the exon 10 of *ATXN3*, leading to a frameshift mutation (Fig. S3B). The sequencing results of 1A revealed that the clone 1A was corrected by adjusting both the mutant CAG allele (CAG80) and normal allele (CAG23) to 13 CAG repeats (Fig. 2F). No indel mutations were detected at sgRNA-binding sites on both alleles in the clone 1A (Fig. 2G). The results of cDNA sequencing revealed that clone 1A was corrected clone, in which the number of CAG repeats was 13 within the exon 10 of *ATXN3* allele (Figs. S4B and S4D). Additionally, we found CBM (GAT) in the *ATXN3* cDNA, indicating that donor was correctly integrated by HDR at *ATXN3* locus (Fig. S4E). Western blot analysis confirmed that the clone 1A was correctly targeted since only a normally sizes ataxin-3 protein from both alleles of corrected *ATXN3* (CAG13) was detected (Fig. 2H).

### The characterization of SCA3-corrected iPSCs

**Figure 3.**
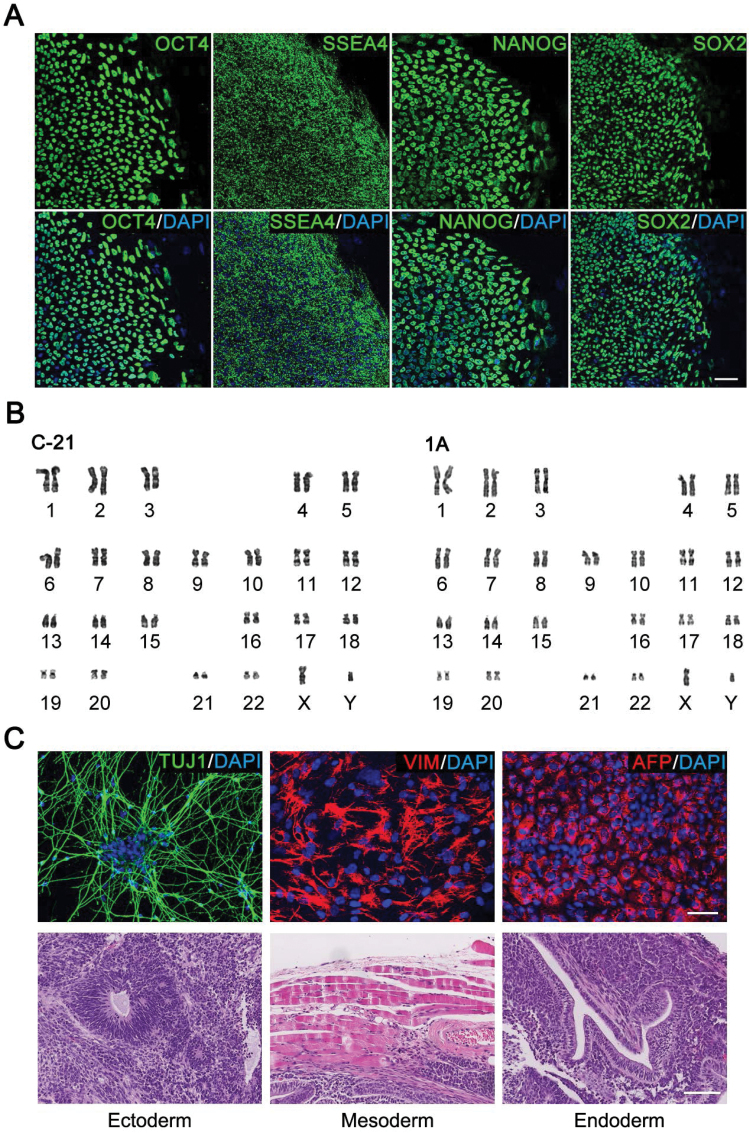
Characterization of genetically corrected SCA3 iPSCs. (A) Immunocytochemistry analysis of genetically corrected iPSCs (represented by clone 1A) detected by human pluripotency markers including OCT4, SSEA4, NANOG and SOX2. Nucleus stained by DAPI. Scale bar represents 50 μm. (B) Karyotyping result showed that no chromosomal abnormalities occurred within the corrected iPSCs. (C) *In vitro* differentiation assay (upper panel) and teratoma assay (lower panel) revealed that corrected iPS cells (represented by clone 1A) could be differentiated into three germ layers- endoderm, mesoderm and ectoderm tissues. Scale bar represents 50 μm in the upper panel and 100 μm in the lower panel.

Next, we investigated whether the *ATXN3*-corrected iPSCs maintained their pluripotency potential. Using an immunohistochemistry approach, we observed strong staining in these corrected cells for human pluripotency markers including OCT4, SSEA4, NANOG and SOX2 (Fig. 3A). An *in vitro* differentiation assay revealed that the corrected iPSCs were able to differentiate into three germ layers- endoderm, mesoderm and ectoderm tissues (Fig. 3C). Upon injection into 2-week-old immunodeficient mice, these corrected iPSCs were capable of forming a teratoma containing three germ layers after 2 months (Fig. 3C). Furthermore, the karyotyping result showed that no major chromosomal abnormalities occurred within these iPSC lines (Fig. 3B).

Next, we performed off-target analysis using whole exome sequencing (WES) and polyacrylamide gel electrophoresis (PAGE). Potential off-target sites for each sgRNA were predicted using the CRISPR design tool from Zhang laboratory (http://crispr.mit.edu/) (Table S1) [12]. WES analysis on two corrected clones (C-21 and 1A) and two SCA3 iPSCs (Pa1 and Pa2-SCA3) revealed that no single nucleotide variants (SNVs) and indels were detected within a 100-bp area from potential off-target sites in the clone C-21 and 1A (Table S2). Additional PAGE analysis also showed that there were no sequence varations in three most similar off-target sites for sgRNA1 and sgRNA2 in the corrected clones (C111, C118 and C206) (Fig. S5).

### Differentiation of SCA3 and genetically corrected iPSCs into cerebellar NSCs

Next, we investigated whether genetic correction led to the reversion of SCA3 disease-associated phenotypes. We chose two corrected clones (C-21 and 1A) combined with SCA3 iPS cell lines (Pa1 and Pa2) and WT iPS cell lines (WT1 and WT2) for subsequent experiments and analysis. We first compared proliferation and apoptosis at the undifferentiated state in the SCA3 iPSCs, corrected iPSCs and WT iPSCs. Given that SCA3 disease was an adult-onset neurodegenerative disease, we speculated that there should be no obvious difference at the undifferentiated state in the corrected, SCA3 and WT iPSCs. As expected, we did not detect any significant differences in cellular morphology, pluripotency (Fig. 3A), apoptosis (Figs. S6A–F) or proliferation (Fig. S6G) among corrected, SCA3 and WT iPSCs.

Previous studies have shown that the cerebellum was one of the most severely affected brain regions in SCA3 patients [2], but little is known about whether neural precursor cells are affected during cerebellar development. Unfortunately, the traditional methods of neural differentiation of iPSCs into cerebellar region-specific neural precursor cells or neural stem cells (NSCs) have not been effectively established. Thus, we tried to develop an effective approach to generate cerebellar NSCs so that we could compare the phenotype changes after genetic correction.

**Figure 4.**
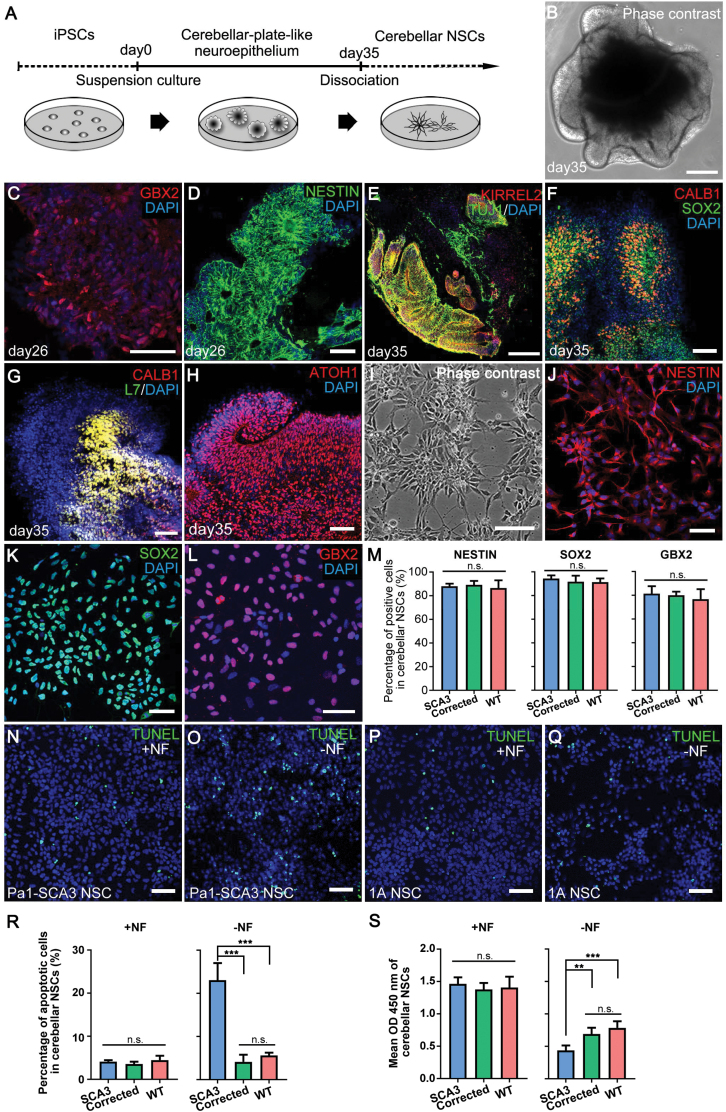
Derivation and characterization of cerebellar NSCs. (A) Procedure of the derivation of cerebellar NSCs from iPSCs. (B) The phase contrast of whole cerebellar tissue induced in 3D culture on day 35. (C and D) Immunostaining for GBX2 and NESTIN on cerebellar-plate-like neuroepithelium on day 26. (E–H) The expression of cerebellar progenitor-specific marker KIRREL2 (E), Purkinje cell markers L7 (G) and CALB1 (F and G), granule cells progenitor marker ATOH1 (H), and neural stem cell marker SOX2 (F) in the cerebellar-plate-like neuroepithelium cultured on day 35. (I) Long-term self-renewing cerebellar neural stem cells generated from differentiated cerebellar tissue. Cerebellar NSCs have an NSC phenotype of rosette-like patterns. (J–L) Expression of cerebellar progenitor-specific markers (GBX2) and neural stem cell markers (SOX2 and NESTIN) in cerebellar NSCs. (M) Percentage of cells positive for GBX2, SOX2 and NESTIN in SCA3, corrected and WT cerebellar NSCs. Each bar represents mean ± SD. Experiments were repeated five times. n.s., not significant. (N–R) TUNEL staining and percentage of apoptotic cells in SCA3, corrected and WT cerebellar NSCs with or without nutritional factors (NF). Each bar represents mean ± SD with five biological replicates. (S) Cell proliferation was assayed in SCA3, corrected and WT cerebellar NSCs with or without NF using a BrdU cell proliferation assay kit. The cell proliferation ratio was measured according to BrdU incorporation by absorbance (Mean OD 450 nm). Each bar represents mean ± SD with five biological replicates. +NF, normal culture condition supplemented with nutritional factors; ‐NF, nutritional factor-withdrawal condition. Samples included SCA3 (Pa1 and Pa2), corrected (C-21 and 1A) and WT (WT1 and WT2) NSCs. The scale bars represent 200 μm (B and E), 100 μm (I) and 50 μm (C, D, F–H, J–L and N–Q). **P < 0.01 and ***P < 0.001 was determined by unpaired *t* test. n.s., not significant.

To effectively generate cerebellar NSCs, we first performed neural differentiation to cerebellar-plate-like neuroepithelium (CPNE) tissue from iPSCs using a serum-free suspension 3D culture approach described previously [21] (Fig. 4A). We found that floating tissue exhibited robust neural differentiation (Fig. 4B) and expressed the neural stem cell markers NESTIN (Fig. 4D) and SOX2 (Fig. 4F) and the neuron-specific marker TUJ1 (as known as β-III tubulin) (Fig. 4E) on days 26 and 35. In addition, we found that the CPNE tissue arisen from the 3D culture system mimicked the development of the cerebellum very well, in which neural tube-like structures were developed in the tissue expressing midbrain-hindbrain marker such as *GBX2*/GBX2, ­cerebellar-plate neuroepithelial specific marker KIRREL2 (as known as NEPH3), Purkinje cell-specific marker L7, CALBINDIN (CALB1) and granule cell progenitor marker ATOH1 (also called MATH1) (Figs. 4C–H). And no obvious morphological differences during neural differentiation to CPNE tissue with 3D culture between the WT, corrected and SCA3 iPSCs (Fig. S7A). Second, we also purified rosette-type, self-renewing cerebellar NSCs from cerebellar neuroepithelium cultured for approximately 35 days according to a previous protocol [22, 23] (Figs. 4A and 4I). More than 80% of the NSCs expressed high levels of the neural stem cell markers NESTIN and SOX2 and the cerebellar precursor marker GBX2 (Figs. 4J–L and S7B), indicating that these cerebellar NSCs faithfully retained the cerebellar regional specification characteristics. We found no obvious morphological differences between the WT, corrected and SCA3 cerebellar NSCs (Figs. 4M and S7B).

Given the prominent clinical and pathological features of cell death in SCA3 disease, we examined apoptosis and proliferation in differentiated cerebellar NSCs using the TUNEL and BrdU cell proliferation assays. Our results indicated that there was no significant difference in apoptosis and proliferation among the WT, corrected and SCA3 cerebellar NSCs under normal culture conditions (with nutritional factor, +NF) (Figs. 4N, 4P, 4R, 4S and S8A). However, under a nutritional factor-withdrawal (without nutritional factor, ‐NF) condition in which cells were cultured in only basal medium (DMEM/F12) without any supplement, the cerebellar NSCs derived from SCA3 iPSCs had a significant (*P* < 0.001) increase (~5-fold) in TUNEL-positive cells when compared to that of the corrected and WT cerebellar NSCs (Figs. 4O, 4Q, 4R and S8A). Similarly, the cerebellar NSCs derived from SCA3 iPSCs also exhibited a significant (*P* < 0.01) decrease in proliferation when compared to that observed in corrected (*P* < 0.01) and WT (*P* < 0.001) cerebellar NSCs under the nutritional factor-withdrawal condition (Fig. 4S). There was no obvious difference between the corrected and WT cerebellar NSCs under the nutritional factor-withdrawal condition (Fig. 4S). These results demonstrated that SCA3 cerebellar NSCs were more vulnerable under nutritional factor-withdrawal condition than corrected cerebellar NSCs.

### Reversion of SCA3 disease-associated phenotypes in corrected cerebellar neurons

**Figure 5.**
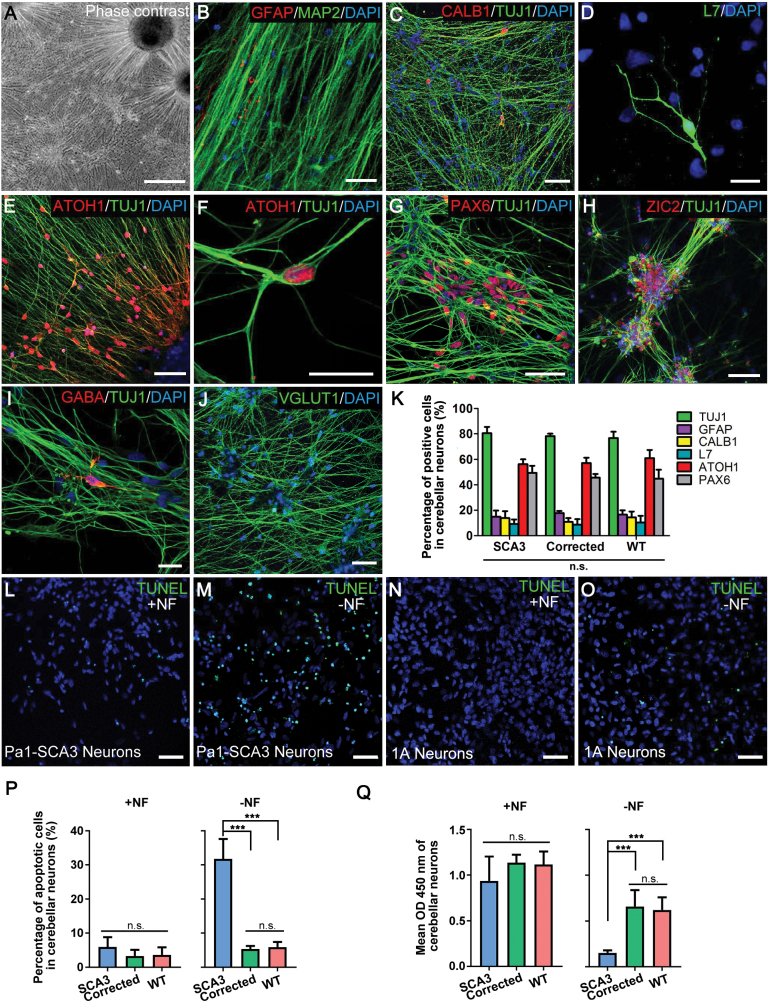
Reversion of SCA3 disease-associated phenotypes in genetically corrected cerebellar neurons derived from cerebellar NSCs.(A) The phase contrast of cerebellar neurons from cerebellar NSCs after prolonged differentiation (>6 weeks) upon growth factor-withdrawal. (B) Expression of MAP2 + neurons and GFAP + glia in 6-week-differentiated neural cultures. (C and D) Immunostaining for L7, CALB1 and TUJ1 on cultured cerebellar neurons (differentiation >6 weeks). (E–H) Immunostaining on 6-week-differentiated neural cultures for granule cells markers (ATOH1, PAX6 and ZIC2). (I and J) The large majority of cerebellar NSC-derived neurons display a glutamatergic phenotype, staining positively for VGLUT1 (J). Only occasional neurons exhibit GABAergic phenotypes (I). (K) Percentage of cells positive for TUJ1, MAP2, GFAP, CALB1, L7, ATOH1 and PAX6 in 6-week-differentiated SCA3, corrected and WT cerebellar neurons. Each bar represents mean ± SD with three biological replicates. (L–P) TUNEL staining and the percentage of apoptotic cells in SCA3, corrected and WT cerebellar neurons after prolonged differentiation (>6 weeks) with or without nutritional factors (NF), which were supplemented for growth and long-term viability of neurons. Each bar represents mean ± SD with three biological replicates. (Q) Cell proliferation was assayed in 3-week-differentiated cultures derived from SCA3, corrected and WT NSCs with or without NF using a BrdU cell proliferation assay kit. Each bar represents mean ± SD with three biological replicates. +NF, normal culture condition supplemented with nutritional factors; ‐NF, nutritional factor-withdrawal condition. Samples included SCA3 (Pa1 and Pa2), corrected (C-21 and 1A) and WT (WT1 and WT2) neurons. The scale bars represent 500 μm (A), 20 μm (B, D, F and I) and 50 μm (C, E, J, G, H and L–O). ***P < 0.001 was determined by unpaired *t* test. n.s., not significant.

To ascertain whether genetic correction was able to reverse the phenotypic abnormalities of SCA3 in cerebellar neurons, we performed region-specific neural differentiation into cerebellar neurons from cerebellar NSCs established from iPSCs. Remarkably, we found that the cerebellar NSC population faithfully retained the cerebellar regional specification and could robustly generate specific cerebellar neurons, consistent with a previous report [23]. After cerebellar NSCs were cultured in differentiation medium for 6 weeks, the cultures exhibited robust neuronal differentiation as most of the differentiated neurons expressed ­neuron-specific marker TUJ1 and microtubule-associated protein-2 (MAP2) (Figs. 5A–C). A small portion of cells also expressed ­glia-specific marker glial fibrillary acid protein (GFAP) (Fig. 5B). After cells were differentiated for 6 weeks, the neuronal cultures exhibited ­region-specific cerebellar lineages, which contained approximately 5% L7 + Purkinje cells (Fig. 5D) and 50% ZIC2 + granule cells (Fig. 5H). In addition, the neuronal cultures were also stained positively for cerebellar region related markers including CALB1 (expressed in Purkinje cells), ATOH1 and PAX6 (expressed in granule cells) (Figs. 5C and 5E–G). Especially, we found that approximately 60% derived neurons displayed a glutamatergic phenotype, staining positively for vesicular glutamate transporter 1 (VGLUT1) (Fig. 5J) and only occasional neurons (~5%) exhibit GABAergic phenotypes (Fig. 5I). We found no apparent differences during differentiation among SCA3, corrected and WT cerebellar neurons (Figs. 5K and S9). To reassure that the cerebellar neurons derived from cerebellar NSCs were electrophysiologically functional, we performed whole-cell patch-clamp recordings from cerebellar neurons differentiated for 6 weeks. The results revealed that the cerebellar neurons could generate multiple action potentials in response to step current injections (Fig. S10A). We found no significant differences on the resting potential (Fig. S10B) and maximum firing frequency (Fig. S10C) among the SCA3, corrected and WT cerebellar neurons.

Subsequently, we examined the apoptotic and proliferative feature of cerebellar neurons derived from the SCA3, corrected and WT cerebellar NSCs. Under normal culture conditions (+NF), we detected 5.9% ± 1.3% TUNEL-positive cells in SCA3 cerebellar neurons, and 3.3% ± 0.8% and 3.6% ± 1.2% TUNEL-positive cells were observed in the corrected and WT cerebellar neurons, respectively (Figs. 5L, 5N, 5P and S8B). Although there was no significant difference in apoptosis among cerebellar neurons under normal culture conditions, we detected higher mean percentage of TUNEL-positive cells in SCA3 cerebellar neurons than corrected or WT cerebellar neurons. But under the nutritional ­factor-withdrawal (‐NF) condition, the percentage of apoptotic SCA3 cerebellar neurons increased to 31.7% ± 2.6%, while the TUNEL-positive cells from corrected and WT cerebellar neurons remained only at 5.3% ± 0.4% and 5.9% ± 0.8% of the cells, respectively (Figs. 5M, 5O, 5P and S8B). We also found that under normal conditions, 3-week-differentiated neural cultures from SCA3, corrected and WT cerebellar NSCs exhibited a similar level of proliferation, but, upon nutritional factor-withdrawal, the SCA3 neural cultures displayed a significant (*P* < 0.001) decrease in cellular proliferation when compared to that in the corrected and WT neural cultures (Fig. 5Q). These results demonstrated that obtaining region-specific cerebellar neurons derived from SCA3 cerebellar NSCs is essential for SCA3 disease modeling and that genetic correction was able to reverse the SCA3 ­disease-associated phenotypic abnormalities in differentiated cerebellar neurons *in vitro*.

### Genetic correction can reverse ataxin-3 spontaneous aggregation in cerebellar neurons

The hallmark of SCA3 diseases is ATXN3-containing aggregates, which have been directly detected in the region-specific brain tissue of patients and are thought to play a critical role in SCA3 pathogenesis [7, 24, 25]. A previous study showed that excitation by l-glutamate in SCA3 iPSC-derived neurons induced formation of SDS-insoluble ataxin-3 aggregates [8]. Considering that the cerebellar region-specific neurons derived from SCA3 iPSCs displayed changes reminiscent of SCA3 disease-associated phenotypes, we hypothesized that these disease-specific and region-specific neurons could be better used for modeling early ATXN3 aggregation *in vitro*.

**Figure 6.**
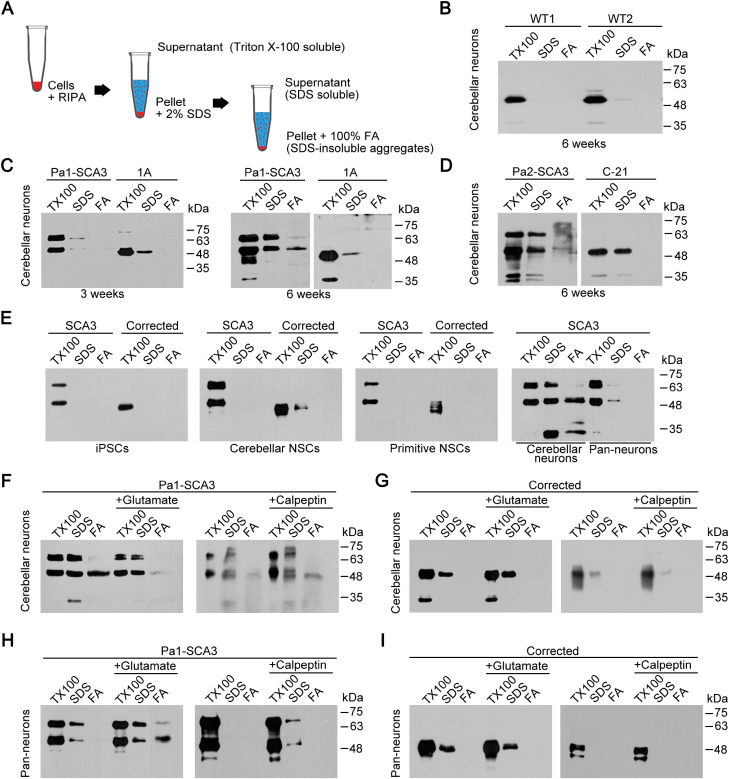
Reversion of ataxin-3 spontaneous aggregation in genetically corrected SCA3 cerebellar neurons. (A) A schematic view of the separation of the cell lysate for investigating the ataxin-3 aggregates. (B-D) Western blot analysis with the ATXN3-specific antibody showed that SDS-insoluble ataxin-3 aggregates appeared in 6-week-differentiated rather than 3-week-differentiated SCA3 cerebellar neurons (C and D), nor in WT (B) and genetically corrected cerebellar neurons (C and D). (E) Ataxin-3 spontaneous aggregation in specific SCA3 cerebellar neurons but not in other cell types such as iPSCs, cerebellar NSCs, pNSCs and pan-neurons derived from pNSCs of SCA3. Strikingly, the hallmark aggregates were not detected in the genetically corrected cells. (F–I) Ataxin-3 aggregation in the presence of the excitatory neurotransmitter l-glutamate (100 μM) or the calpain inhibitor calpeptin (100 μM) in SCA3 cerebellar neurons and pan-neuronal cells (F, H). Remarkably, SDS-insoluble ataxin-3 aggregates were not detected in either the genetically corrected cerebellar neurons or the pan-neuronal cells (G, I). TX100, Triton-X-100; FA, formic acid. *n* ≥ 3 individual experiments.

To detect SDS-insoluble ataxin-3 aggregates, we separated cell lysates into fractions based on different detergent solubilities (Triton-X-100 soluble fraction, SDS-soluble fraction and SDS-insoluble fraction) (Fig. 6A) [8]. We found that mature SCA3 cerebellar neurons differentiated for 6 weeks could spontaneously form SDS-insoluble aggregates without induction by l-glutamate (Figs. 6C and 6D). However, no SDS-insoluble ataxin-3 aggregates were observed in mature WT and corrected cerebellar neurons (Figs. 6B–D). We also could not detect any signal of SDS-insoluble ataxin-3 aggregates in immature SCA3 or corrected cerebellar neurons (Fig. 6C). Then, we asked whether this spontaneous ataxin-3 aggregation could be evoked in SCA3 pan-neuronal cells or in SCA3 iPSCs or NSCs. In other words, we wondered whether spontaneous ataxin-3 aggregate formation is restricted only to SCA3 ­disease-specific cerebellar neurons. Then, we generated primitive neural stem cells (pNSCs) from iPSCs, which retained the primitive state of neural differentiation without region specification, and further derived pan-neurons from the pNSCs [26]. Importantly, we found that only SCA3 cerebellar neurons formed spontaneous SDS-insoluble aggregates (Fig. 6E), while no ataxin-3 aggregates were observed in the SCA3-corrected cells (Figs. 6C–E), indicating that genetic correction could reverse formation of ataxin-3 aggregates in cerebellar neurons in early stages of SCA3 pathogenesis.

Next, we investigated whether the formation of ataxin-3 spontaneous aggregates in cerebellar neurons involved calpain or caspase, which was thought to cleave ataxin-3 to induce aggregate formation [7, 8, 27, 28]. The activity of calpain and caspase relies on intracellular Ca^2+^, the level of which can be elevated by treatment with l-glutamate (100 μM) in differentiated neurons (Fig. S11A). A previous study showed that the excitation by l-glutamate in SCA3 iPSC-derived neurons induced formation of SDS-insoluble ataxin-3 aggregates depended on calpain [8]. In SCA3 pan-neurons, we indeed found that L-glutamate (100 μM) induced formation of SDS-insoluble ataxin-3 aggregates (Fig. 6H), which is consistent with previous finding [8]. In addition, no aggregates were detected in the presence of the calpain inhibitor calpeptin (100 μM) (Fig. 6H). However, in SCA3 cerebellar neurons, we detected a decrease in the aggregates in the presence of l-glutamate (100 μM) and the formation of more aggregates when treated with calpeptin (100 μM) or a pan-caspase inhibitor Z-VAD-FMK (10 μM) (Figs. 6F, S11B and S11E). This result indicated that the formation of ataxin-3 aggregates in SCA3 cerebellar neurons is different from that of SCA3 pan-neurons. Ca^2+^-related pathways are involved in the formation of spontaneous ataxin-3 aggregates in SCA3 cerebellar neurons, but inhibition of calpain or caspase cannot prevent spontaneous ataxin-3 aggregation during early SCA3 pathogenesis.

**Figure 7.**
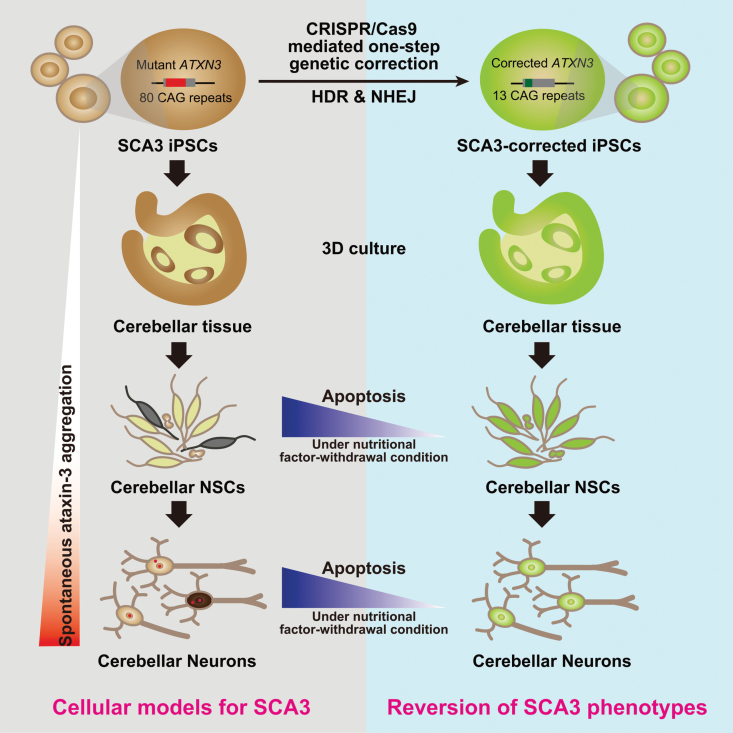
CRISPR/Cas9-mediated genetic correction reverses Spinocerebellar ataxia 3 disease-associated phenotypes in differentiated cerebellar neurons.

Importantly, SDS-insoluble ataxin-3 aggregates were not detected in WT and genetically corrected cerebellar neurons or corrected pan-neuronal cells treated with l-glutamate or calpeptin, suggesting that genetic correction can reverse ataxin-3 aggregation in neurons during early SCA3 pathogenesis (Figs. 6G, 6I and S11C). Additionally, no SDS-insoluble ataxin-3 aggregates were observed in immature SCA3 cerebellar neurons treated with l-glutamate (Fig. S11D). Next, when we performed immunocytochemistry analysis on this early aggregation phenotype, we found no visible ataxin-3 inclusion bodies or macro-aggregates in SCA3 and corrected cerebellar neurons (Fig. S11F), which was not surprising because inclusion bodies or macro-aggregates have always been shown to emerge in the late disease stages in SCA3 patients [29, 30]. In sum, our findings suggest that iPSC-derived disease-specific and region-specific cerebellar neurons can provide unique cellular models for the study of SCA3 pathogenesis, and genetic correction can reverse the SCA3 disease-associated phenotypes *in vitro* (Fig. 7).

## Discussion

Genetic manipulation in human iPSCs has been a critical step in the elucidation of gene functions and molecular pathways underlying disease mechanisms. Genetic correction of trinucleotide repeat disorders in iPSCs has become a powerful tool for illustrating relevant disease mechanisms *in vitro* [31–33]. In this study, we established SCA3 disease-specific iPSCs from urine-derived cells of the SCA3 patients and achieved one-step genetic correction in SCA3-specific iPS cells using the CRISPR/Cas9 system.

“Compared to other strategies about genetic correction of trinucleotide repeat disorders, there are several advantages of the intron- and exon-based strategies we developed. (i) In previous studies, the donor plasmid always contains a removable cassette with selection markers (such as resistance genes) to promote the screening [34–36]. To avoid the possible effects of the introduced cassette, additional steps were necessary to remove the cassette in previous studies. In our study, we used the dual-sgRNA and a donor plasmid without the selection cassette for genetic correction, which is more efficient and convenient. (ii) Although several studies have applied CRISPR/Cas9 system to directly delete the trinucleotide repeat region, it also raised concerns about whether these strategies would interfere the normal function of target genes due to the alteration of protein sequence after genetic correction [37]. Since we applied the intron- and exon-based strategies for genetic correction of SCA3 disease, our genetically corrected cells expressed full-length ataxin-3 protein to ensure the normal function of ataxin-3 protein. (iii) In previous studies, the homologous arms always reached several kb in length, which increased the difficulty of the plasmid construction, transfection and the screening [32, 34]. In our study, the length of the homologous arms was approximately 1 kb on both sides in donor-2201 and only 81 bp (left) and 406 bp (right) in donor-608, which are significantly shorter than that used in previous studies [32, 34]. Short homologous arms ensure a simple and direct screening assay by PCR and may be further adapted for genetic correction under other conditions. Thus, the targeting strategy for genetic repair of SCA3 diseases may be applied to other trinucleotide disorders if applicable.”

One of the major difficulties in studying SCA3 pathogenesis is the absence of effective tools to study cerebellar neurodegeneration *in vivo*. *In vitro* differentiation of disease-specific iPSCs provides a systematic platform to investigate human CNS disease [19]. Compared to previous neural differentiation for the study of the SCA3 disease, here, we first performed a cerebellar region-specific differentiation combined with 3D neural culture to achieve quantitative cell populations—cerebellar NSCs and neurons. The cerebellar NSCs that we established faithfully retained the cerebellar regional specification and could robustly generate specific cerebellar neurons. These cerebellar region-specific cell populations not only support a powerful tool for SCA3 disease modeling *in vitro* but also are of great significance in cell therapy and drug discovery for SCA3 disease. In addition, other brain regions were also affected by SCA3 disease besides the cerebellar damage. Our methods of cerebellar neuron differentiation in this paper provide a new way to obtain other region-specific neurons for further SCA3 study. It is also meaningful to utilize similar methods to study neurodegenerative diseases such as Huntington’s and Alzheimer’s disease which also cause damage in specific brain region like SCA3.

So far, there are several hypotheses for SCA3 pathogenesis, including toxic ataxin-3 fragments [38], transcriptional deregulation [39], mitochondrial dysfunction [40], deficiency of p53 activity [41], etc. To decipher the pathogenic mechanism, we systematically compared SCA3 disease-specific iPSCs with corrected and WT iPSCs and subsequently performed a cerebellar ­region-specific differentiation to compare these cells at various differentiated stages and cell types, since all types of the differentiated SCA3-corrected cells shared an isogenic background. Our experiments verified that SCA3 cerebellar NSCs and neurons were more vulnerable under the nutritional factor-withdrawal (‐NF) condition than WT cerebellar NSCs and neurons. Genetic correction was able to reverse the SCA3 disease-associated phenotypic abnormalities in differentiated cerebellar neurons.

Furthermore, hallmark of SCA3 diseases is ATXN3-containing aggregates, which are thought to play a critical role in the disease’s pathology [7, 24, 25]. In our study, for the first time, we observed spontaneous ataxin-3 aggregates specifically in mature cerebellar neurons differentiated from SCA3 iPSCs rather than in SCA3 pan-neurons, SCA3 iPSCs or NSCs. Given that derived SCA3 cerebellar neurons were prone to apoptosis, these results provide the first explanation for why cerebellar region-specific damage occurs in SCA3 disease. Our study showed that the spontaneous ataxin-3 aggregation in SCA3 cerebellar neurons was essentially different from excitation-induced ataxin-3 aggregation in SCA3 pan-neurons. The formation of spontaneous ataxin-3 aggregates in SCA3 cerebellar neurons is effected by Ca^2+^-related pathways but may not be caused by calpain and caspase, which had been thought to play important roles in ataxin-3 aggregation [7, 8, 27, 28]. This contradictory result may be explained by the use of cerebellar region-specific neurons in our experiments, which contains many glutamatergic neurons. Importantly, SDS-insoluble ataxin-3 aggregates were not detected in genetically corrected cerebellar neurons and pan-neuronal cells. In our study, we demonstrate that genetic correction can indeed reverse SCA3 disease progression, giving new promise for potential therapeutic applications in the future.

In summary, we established efficient methods for one-step genetic correction in SCA3 iPSCs using CRISPR/Cas9 technology and subsequently developed SCA3 disease modeling in cerebellar region-specific and disease-specific differentiated neurons. This work provides unique cellular models for studying the pathogenic mechanism of SCA3 disease and demonstrated that genetic correction can reverse SCA3 disease progression *in vitro*.

## Research limitations

In this manuscript, we have established SCA3 ­disease-specific iPSCs from SCA3 patients and achieved one-step genetic correction in SCA3 iPSCs using the CRISPR/Cas9 system. Although we have developed the simple and rapid gene targeting methods—the intron-based and exon- based strategies—for SCA3 iPSCs, the overall HDR editing efficiency is relatively low, which needs to be further optimized. Considering the latest advances of recently reported prime editing (PE) systems [42, 43], developing novel strategies using PE systems for SCA3 gene editing may provide an alternative choice. In addition, the detailed SCA3 pathogenesis remains to be elucidated, although several hypotheses for SCA3 pathogenesis were proposed, such as toxic ataxin-3 fragments [38], transcriptional deregulation [39], mitochondrial dysfunction [40], deficiency of p53 activity [41], dysfunction in Purkinje cells [44], etc. In this study, we have established SCA3-corrected iPSCs with an isogenic background and derived disease-specific and region-specific cerebellar neurons from these iPSCs, which provide unique cellular models for studying SCA3 pathogenesis *in vitro*. Given that the exact mechanism of SCA3 remains unsolved, it is essential to decipher the pathogenesis of SCA3 disease through in-depth analysis of the phenotypes in molecular and cellular levels, such as degeneration and potassium channel dysfunction in Purkinje cells, mitochondrial dysfunction and oxidative stress. And it is also important to establish different region-specific neurons derived from SCA3 and genetically corrected iPSCs for more precisely cellular modeling of SCA3.

## Materials and methods

### Generation of patient-specific iPS cells

The urinary samples of donors were collected for non-viral serum-free iPS generation as described previously [20]. Human iPSCs were cultured on either Geltrex (Gibco) in Essential 8 medium (Invitrogen) or mouse embryonic fibroblasts (MEFs) inactivated by mitomycin C in human iPSC medium containing DMEM/F12 medium (Sigma) supplemented with 20% knockout serum replacement (Gibco), 2 mM l-glutamine, 1% ­2-mercaptoethanol, 1% nonessential amino acids, 100 U/mL penicillin, 100 μg/mL streptomycin (all from Millipore) and 10 ng/mL basic fibroblast growth factor (bFGF, R&D).

### Donors and sgRNA expression vector construction

For donor vector construction, the DNA was synthesized and ligated into a pUC18 cloning vector (Biomed). Construction of sgRNA expression vector was performed as previously described [45, 46]. Briefly, the annealed oligonucleotides for each sgRNA were cloned into a pGL3-sgRNA expression vector driven under U6 promoter for human cell transfection. For details on experimental procedures, see Supplementary Information.

### SCA3 iPSCs transfection and screening

One day before transfection, at least 3 × 10^6^ iPS cells/well were planted on Geltrex-coated six-well plate in Essential 8 medium supplemented with 10 μM ROCK inhibitor Y-27632 (Sigma). For genetic correction in SCA3 iPSCs, cells were transfected with 1 µg of each sgRNA plasmid, 2 µg of donor DNA plasmid, and 1 µg of pCas9-GFP plasmid (Addgene number: 44719) by Lipofectamine® LTX reagent (Invitrogen) according to the manufacturer’s protocol. After 1 day, iPS cells were sorted by FACS (FACSAriaIII, BD) and 3000 cells/well cultured on ­feeder-coated six-well plate in iPSC medium supplemented with Y-27632 (first 2 day) for another 2 weeks until single clones could be picked for genotyping. Half of the single clones were placed into a 0.2-mL tube for genomic DNA extraction, and the other half was placed into a 96-well plate for maintenance. Genomic DNA from individual iPS cell clones were quickly extracted by DNA extraction solution 1.0 (BuccalAmp). PCR and 3% agarose gel electrophoresis were used to detect genomic modification. The expected PCR band was purified for further sequencing analysis.

### Immunocytochemistry analysis

Cultures on the coverslips were fixed with 4% paraformaldehyde (Sigma) in PBS (pH 7.4) for 20 min at RT, then washed with PBST (0.5% Triton-X-100 in PBS) for 10 min twice and blocked with 5% horse serum (Sigma) for 1 h. Samples were incubated with the primary antibodies in dilution buffer (0.2% Triton-X-100 and 0.5% bovine serum albumin in PBS; BSA, from Sigma) at 4°C overnight, washed three times with PBST and incubated with secondary antibody in dilution buffer conjugated to the fluorescent labels Alexa 568 or 488 (1:200; Invitrogen) at RT for 1 h, washed three times and counterstained the nuclei with DAPI (4ʹ, 6-diamidino-2-phenylindole, Sigma).

**Table:** 

Human primary antibodies used in this study			
Antibody	Company	Host species	Dilution
SOX2	Proteintech	Rabbit	50
OCT4	Proteintech	Rabbit	200
SSEA4	Cell Signaling	Mouse	200
NANOG	Proteintech	Rabbit	100
KIRREL2	Proteintech	Rabbit	100
MAP2	Origene	Mouse	200
MAP2	Proteintech	Rabbit	200
TUJ1	Beyotime	Mouse	800
CALB1	Proteintech	Rabbit	200
ATXN3	Millipore	Mouse	500
ATXN3	Origene	Rabbit	80
POLYGLUTAMINE	Millipore	Mouse	1000
GFAP	Millipore	Rabbit	100
NESTIN	Proteintech	Rabbit	100
ATOH1	Proteintech	Rabbit	200
VIM	Proteintech	Rabbit	200
AFP	Proteintech	Rabbit	100
GBX2	Proteintech	Rabbit	50
PAX6	Proteintech	Rabbit	100
L7	SantaCruz	Mouse	200
ZIC2	Sangon	Rabbit	50
GABA	Sigma	Rabbit	400
VGLUT1	Proteintech	Rabbit	50

### Cerebellar NSCs and pNSCs derivation from iPSCs

To generate cerebellar NSCs, we first performed neural differentiation to cerebellar-plate-like neuroepithelium from iPSCs using serum-free suspension culture previously described [21] with slight modifications. Next, we purified a rosette-type, ­self-renewing CPNE stem cells population from cerebellar neuroepithelium cultured for about 35 days according to previous protocol [22, 23]. Neural tube-like structures developed in the EBs outgrowth expressed CPNE-specific marker KIRREL2 (Fig. 4E). These structures were mechanically separated with needle and dissociated to single cells with 0.25% Trypsin/EDTA (Millipore). For pNSCs derivation, we performed a rapid neural induction using PSC Neural Induction Medium (Invitrogen) as a manufacture’s protocol. Briefly, iPSCs were cultured in PSC Neural Induction Medium containing of Neurobasal medium supplemented with Neural Induction Supplement on Geltrex. For details on experimental procedures, see Supplementary Information.

### Differentiation of NSC lines to neurons

Neuronal differentiation was induced as previously described [22] by removing the growth factors bFGF and EGF from the media. Cells were cultured on poly-ornithine/laminin coated dishes or coverslips in differentiation medium containing DMEM/F12 with Neurobasal mixed at a 1:1 ratio, N2 supplement (1:100), B27 supplement (1:100), cAMP (300 ng/mL, Sigma). The pan-neurons were derived from non-region-specific pNSCs. The cerebellar neurons were derived from cerebellar NSCs, and when performed cerebellar neuron differentiation, retinoic acid was added to the media in the form of vitamin A provided in the B27 supplement (Invitrogen). Analyses were usually conducted in neuronal cultures differentiated 6–8 weeks.

### TUNEL assay

TUNEL assay was performed with TUNEL Apo-Green Detection Kit (Biotool) as manufacture’s protocol. For iPSCs and NSCs, cells were seeded equally on coverslips in four-well plate in normal medium for 1 day. For nutritional factors withdrawal condition, iPSCs were then cultured in E8 basal medium without any supplement. And NSCs were then cultured in DMEM/F12 medium without any supplement. Cells were under the withdrawal condition for 24 h before fixation with 4% paraformaldehyde (Sigma) for 1 h. After washing with DPBS (GIBCO), cells were permeablized in PBS with 0.2% Triton-X-100 (Sigma) for 5 min at room temperature (RT), then equilibrated with equilibration buffer for 10 min at RT. Subsequently, cells were incubated with a TUNEL reaction mixture containing Apo-Green Labeling Mix and recombinant terminal deoxynucleotidyl transferase (rTdT) for 1 h at 37°C. Cells were counterstained with DAPI. For neurons, cells were differentiated for 6 weeks in differentiation medium. For nutritional ­factor-withdrawal condition, cells were then cultured in DMEM/F12 with Neurobasal medium mixed at a 1:1 ratio without any supplement for 48 h. Subsequently, TUNEL assay was performed as described above.

### BrdU cell proliferation assay

Cell proliferation assay was performed with BrdU Cell Proliferation Assay Kit (Cell Signaling Technology) following manufacture’s protocol. For iPSCs, cells were seeded equally on Geltrex in 96-well plate in E8 medium for 1 day. For nutritional factor-withdrawal condition, cells were then cultured in E8 basal medium without any supplement. After 24 h, 10 µM BrdU was added to the plate and cells were incubated for 4 h. Then cells were fixed for 30 min and stained with antibody for BrdU at RT for 1 h. HRP-conjugated secondary antibody and TMB substrate were used to detect the BrdU incorporation by measured absorbance at 450 nm. For NSCs, cells were seeded on poly-ornithine/laminin coated 96-well plate in NSCs medium for 1 day. For nutritional factor-withdrawal condition, cells were then cultured in DMEM/F12 medium without any supplement. The BrdU incorporation and detection was performed like iPSCs proliferation assay described above. For differentiated cultures, cells were differentiated for 2 weeks in differentiation medium. For nutritional factor-withdrawal condition, cells were then cultured in DMEM/F12 with Neurobasal medium mixed at a 1:1 ratio without any supplement for 1 week. Subsequently, BrdU assay was performed as described above.

### Compliance with ethical guidelines

SCA3 patients and healthy individuals in this manuscript have signed a written informed consent for donating isolated epithelial cells from urinary samples for stem cell research. The human subject protocol was reviewed and approved by the institutional committee for human ethics at Institute of Biophysics, Chinese Academy of Sciences. All procedures concerning the care and use of animals were performed according to IACUC approved protocols.

### Off-target analysis

First, we identified potential off-target sites using the CRISPR design tool (http://crispr.mit.edu) with the synthetic 20-nt sgRNA sequence plus 5ʹ-NGG “proto-spacer adjacent motif” (PAM) sequence. Next, off-target analysis was determined by WES and PAGE. For WES analysis, the off-target was detected by whether the variants were located at potential off-target site. The detailed analysis was performed according to previous study [47, 48]. PAGE was performed on detection of the three most similar off-target sites for sgRNA. The selected off-target sites were amplified with PCR reaction followed with standard PAGE assay as described [49].

### Western blot analysis

Cultures were washed three times with ice-cold DPBS and immediately frozen in liquid N2 followed by lysed in lysis buffer containing 50 mM Tris-HCl (Ph 7.4), 150 mM NaCl, 1% NP-40, 0.1% SDS, 1 mM EDTA, 10 μg/mL leupeptin and 10 μg/mL aprotinin, 10 μg/mL pepstatin and 10 mM phenylmethylsulfonyl fluoride (PMSF). Then cellular debris were pelleted by centrifugation at 12,000 *g* for 15 min at 4°C, and the concentration of protein in the supernatant was estimated by the BCA method. The samples were mixed with loading buffer and boiled for 5 min for denaturation. For western blot analysis, 40 μg of protein was loaded in each well and separated on 10% SDS-polyacrylamide gels (Bio-Rad equipment). After electrophoresis, the protein was electrically transferred onto nitrocellulose membranes (0.45 mm) at 200 mA for 2 h. Membranes were then blocked in 0.5% BSA in Tris-buffered saline with 0.1% Tween 20 (TBST), and incubated with primary antibodies at 4°C overnight, washed three times in TBST, the blots were incubated with horseradish ­peroxidase-conjugated secondary antibodies (1:2000; GenStar) at RT for 1 h. Then the membrane was rinsed with TBST, followed by detection using enhanced chemiluminescence. The iPSCs were cultured in feeder-free condition for Western blot analyses. The mouse anti-ATXN3 monoclonal antibody (MAB5360) and mouse anti-polyQ monoclonal antibody (clone 1C2) (MAB1574) were purchased from Millipore. The other rabbit anti-ATXN3 polyclonal antibody (TA327022) was purchased from Origene. The anti-actin antibody was obtained from GenStar.

### Ataxin-3 aggregation analysis

The ataxin-3 aggregation detection was performed as previously described [8]. Briefly, cells cultured in six-well plates were washed three times with ice-cold DPBS and immediately frozen in liquid N2 followed by lysis in RIPA buffer containing 50 mM Tris, 150 mM NaCl, 25 mM EDTA, and 0.2% Triton-X-100. For ­l-glutamate stimulation, neurons (cerebellar neurons or ­pan-neurons) were washed three times with 2 mL balanced salt solution (BSS) containing 25 mM Tris, 120 mM NaCl, 15 mM glucose, 5.4 mM KCl, 1.8 mM CaCl_2_, 0.8 mM MgCl_2_, pH 7.4. Then cells were treated with 100 μM ­l-glutamate (Sigma) in BSS for 30 min. For inhibition studies, neurons were incubated in differentiation medium with 100 μM calpeptin (Selleck) or 10 μM Z-VAD-FMK (Selleck) for 24 h. After treatment, cells were washed three times and immediately frozen in liquid N2 followed by lysis in RIPA buffer. For aggregation analysis of ataxin-3, lysates were separated to fractions ­(Triton-X-100-soluble fraction, SDS-soluble fraction and FA-resoluble aggregates [50]) for western blot detection with mouse anti-ATXN3 monoclonal antibody (MAB5360) or rabbit anti-ATXN3 polyclonal antibody (TA327022).

### Data availability

All data generated during this study are included in this article.

## Supplementary Material

lnac020_suppl_Supplementary_Material
