# Supplementary material for: CRISPR/Cas9-mediated genetic correction reverses spinocerebellar ataxia 3 disease-associated phenotypes in differentiated cerebellar neurons
Source: Life Med. 2022 Jun 29;1(1):27–44. doi: 10.1093/lifemedi/lnac020 (PMC11749335; doi:10.1093/lifemedi/lnac020)
Supplement: lnac020_suppl_Supplementary_Material [file lnac020_suppl_Supplementary_Material.pdf]

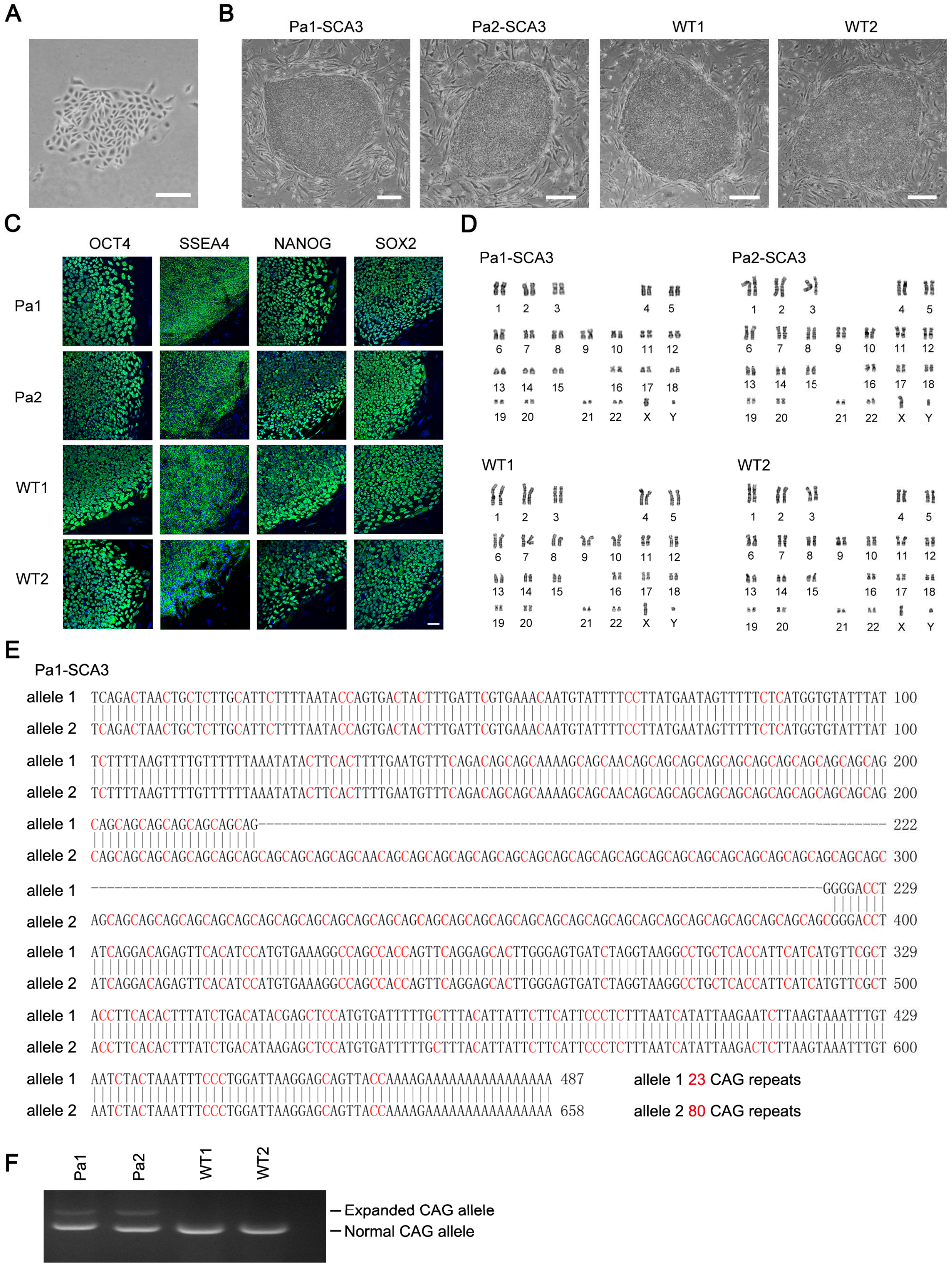

Figure S1. Characterization of the iPS cell lines from the SCA3 patients and WT controls, Related to Figure 1 and 2. (A) The phase contrast of urine-derived cells. (B) The phase contrast of different iPS cell lines generated from SCA3 patients and WT controls using an episomal-based protocol. (C) Immunohistochemistry analysis revealed strong pluripotent marker expression in iPS cells. Pluripotency markers used in this study include OCT4, SSEA4, SOX2 and NANOG. (D) Karyotyping analysis demonstrated normal karyotype in the SCA3 and WT iPS cell lines. (E) Sequencing of the Pa1-SCA3 iPS cell line confirms 80 CAG repeats in the mutant allele and 23 CAG repeats in the normal allele. (F) PCR analysis was performed to assess the length of CAG repeats. The scale bars represent 200 μm (A and B) and 50 μm (C).

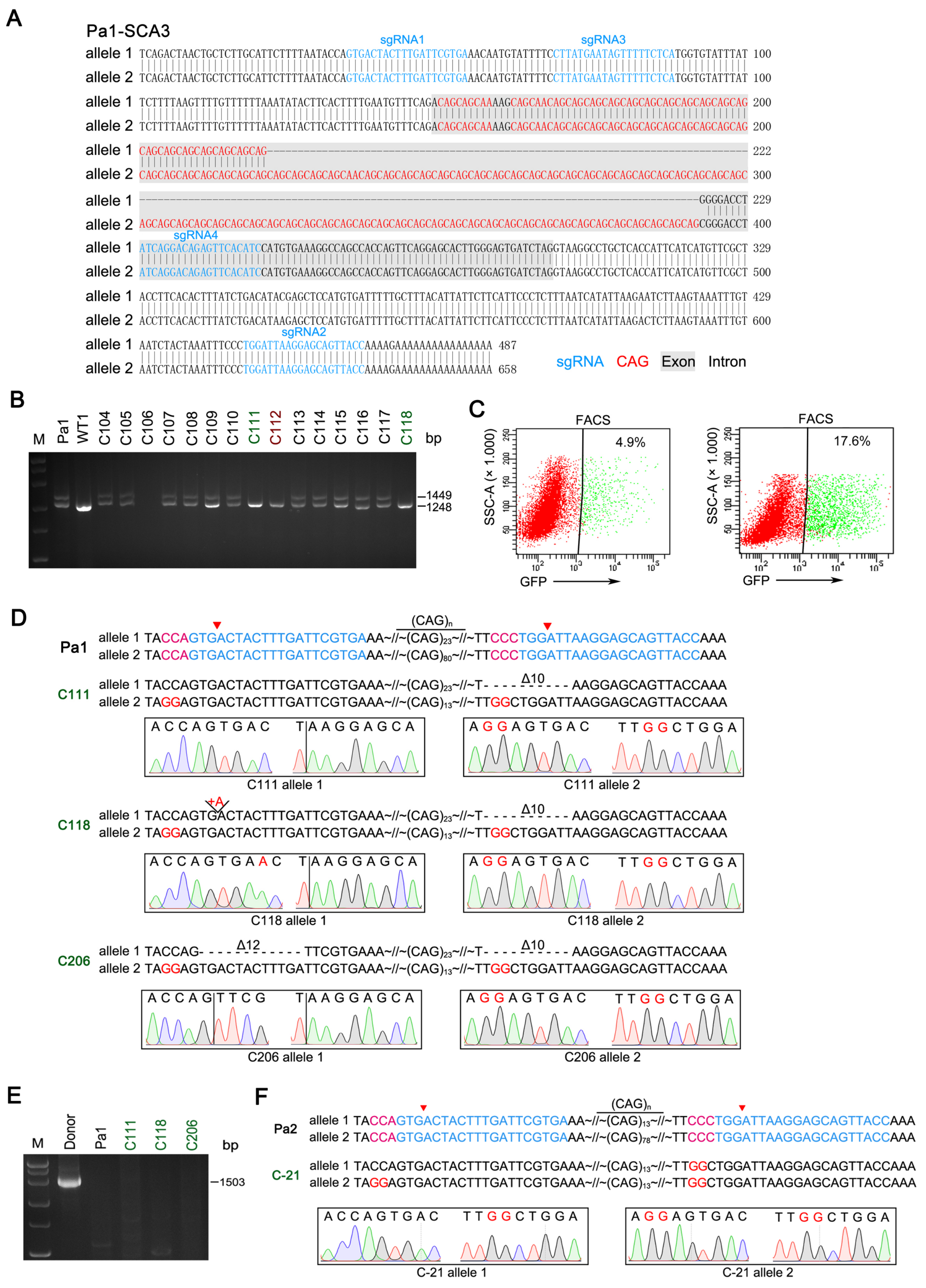

Figure S2. The location of the sgRNA target sites and sequencing results of C111, C118, C206 and C-21, Related to Figure 1. (A) The location of the sgRNA target sites. Both *ATXN3* alleles can be recognized and

edited by the CRISPR/Cas9 system. (B) PCR-based screen of genetically corrected iPSC clones after gene targeting in Pa1-SCA3 iPSCs using primer pair P1. (C) The ratio of GFP-positive iPS single cells by FACS after 1 day of transfection, usually ranging from 5% and up to 17.6%. (D) Sequencing analysis of the Pa1-SCA3 genetically corrected clones (C111, C118, C206) near the CAG repeat region including the sgRNA target sites (blue) and PAM (magenta). (CAG)n, CAG repeat number; red triangles, cleavage sites; ∆10, delete 10 base pairs; +A, insert an adenine. (E) Exclusion of donor DNA random integration into the chromosomes by PCR using primer pair D2201-T in corrected iPS cell lines. (F) Sequencing analysis of the Pa2-SCA3 genetically corrected clones C-21 near the CAG repeat region including the sgRNA target sites (blue) and PAM (magenta).

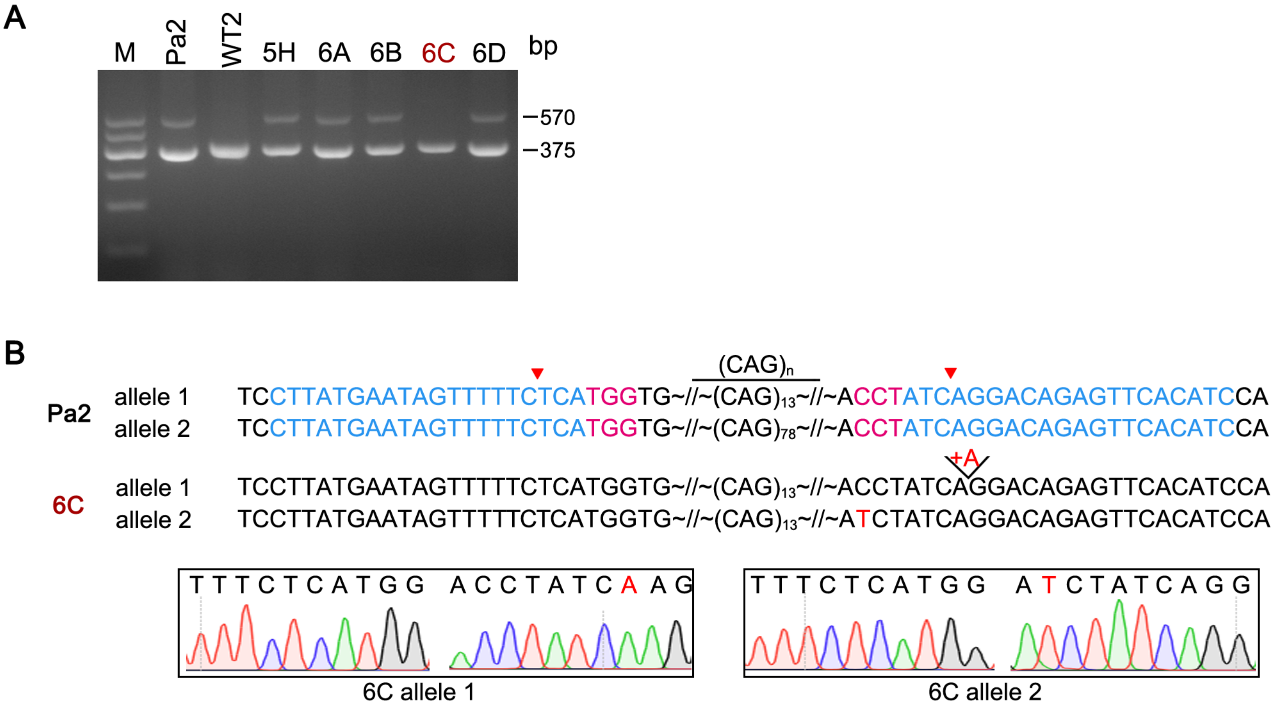

Figure S3. Gene targeting on Pa2-SCA3 iPSCs using the "exon-based strategy". Related to Figure 2. (A) PCR-based screen of genetically corrected iPSC clones after gene targeting in Pa2-SCA3 iPSCs using primer pair P3. (B) Sequencing analysis indicated that clone 6C was an uncorrected iPSC clone that contains an adenine insertion on the sgRNA4 target site in the exon 10 of *ATXN3*, leading to a frameshift mutation.

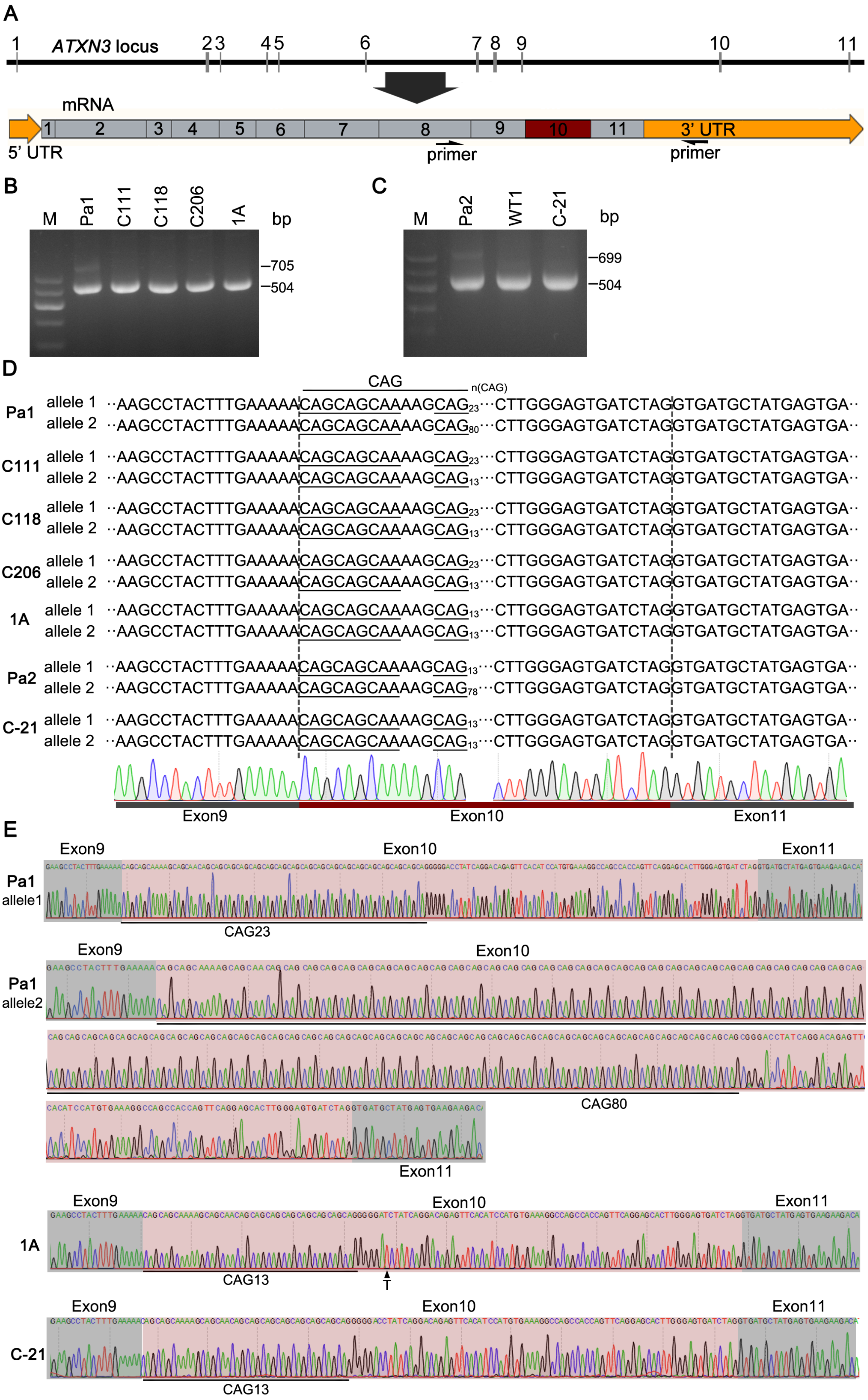
A
ATXN3 locus
1
2 3
4 5
6
7 8
9
10
11
mRNA
5' UTR
3' UTR
primer
primer
B
M
Pa1
C111
C118
C206
1A
bp
705
504
C
M
Pa2
WT1
C-21
bp
699
504
D
CAG
n(CAG)
Pa1 allele 1 ··AAGCCTACTTTGAAAAACAGCAGCAAAAGCAG23···CTTGGGAGTGATCTAGGTGATGCTATGAGTGA··
allele 2 ··AAGCCTACTTTGAAAAACAGCAGCAAAAGCAG80···CTTGGGAGTGATCTAGGTGATGCTATGAGTGA··
C111 allele 1 ··AAGCCTACTTTGAAAAACAGCAGCAAAAGCAG23···CTTGGGAGTGATCTAGGTGATGCTATGAGTGA··
allele 2 ··AAGCCTACTTTGAAAAACAGCAGCAAAAGCAG13···CTTGGGAGTGATCTAGGTGATGCTATGAGTGA··
C118 allele 1 ··AAGCCTACTTTGAAAAACAGCAGCAAAAGCAG23···CTTGGGAGTGATCTAGGTGATGCTATGAGTGA··
allele 2 ··AAGCCTACTTTGAAAAACAGCAGCAAAAGCAG13···CTTGGGAGTGATCTAGGTGATGCTATGAGTGA··
C206 allele 1 ··AAGCCTACTTTGAAAAACAGCAGCAAAAGCAG23···CTTGGGAGTGATCTAGGTGATGCTATGAGTGA··
allele 2 ··AAGCCTACTTTGAAAAACAGCAGCAAAAGCAG13···CTTGGGAGTGATCTAGGTGATGCTATGAGTGA··
1A allele 1 ··AAGCCTACTTTGAAAAACAGCAGCAAAAGCAG13···CTTGGGAGTGATCTAGGTGATGCTATGAGTGA··
allele 2 ··AAGCCTACTTTGAAAAACAGCAGCAAAAGCAG13···CTTGGGAGTGATCTAGGTGATGCTATGAGTGA··
Pa2 allele 1 ··AAGCCTACTTTGAAAAACAGCAGCAAAAGCAG13···CTTGGGAGTGATCTAGGTGATGCTATGAGTGA··
allele 2 ··AAGCCTACTTTGAAAAACAGCAGCAAAAGCAG78···CTTGGGAGTGATCTAGGTGATGCTATGAGTGA··
C-21 allele 1 ··AAGCCTACTTTGAAAAACAGCAGCAAAAGCAG13···CTTGGGAGTGATCTAGGTGATGCTATGAGTGA··
allele 2 ··AAGCCTACTTTGAAAAACAGCAGCAAAAGCAG13···CTTGGGAGTGATCTAGGTGATGCTATGAGTGA··
Exon9
Exon10
Exon11
E
Pa1 allele1
Exon9
Exon10
Exon11
CAG23
Pa1 allele2
Exon9
Exon10
CAG80
Exon11
1A
Exon9
Exon10
Exon11
CAG13
T
C-21
Exon9
Exon10
Exon11
CAG13

Figure S4. Sequencing analysis of *ATXN3* cDNA in genetically corrected iPSCs. Related to Figure 1 and 2. (A) A schematic view of *ATXN3* cDNA sequencing. Gray boxes represent exons at the *ATXN3* locus or mRNA. (B and C) PCR analysis on *ATXN3* cDNA to assess the genetic correction in different iPS cell lines. (D) Sanger sequencing on exon 10 and its adjacent region of *ATXN3* cDNA in different iPS cell lines. The sequencing results indicated that Pa1-SCA3 genetically corrected clones (C111, C118, C206 and 1A) and Pa2-SCA3 genetically corrected clone C-21 had normal length of CAG repeats at both *ATXN3* alleles. (E) Sequencing results about *ATXN3* cDNA on exon 10 and its adjacent region of Pa1-SCA3, 1A and C-21 clones. The exon 10 of clone 1A had a same-sense mutation (GAC to GAT), which was introduced by donor.

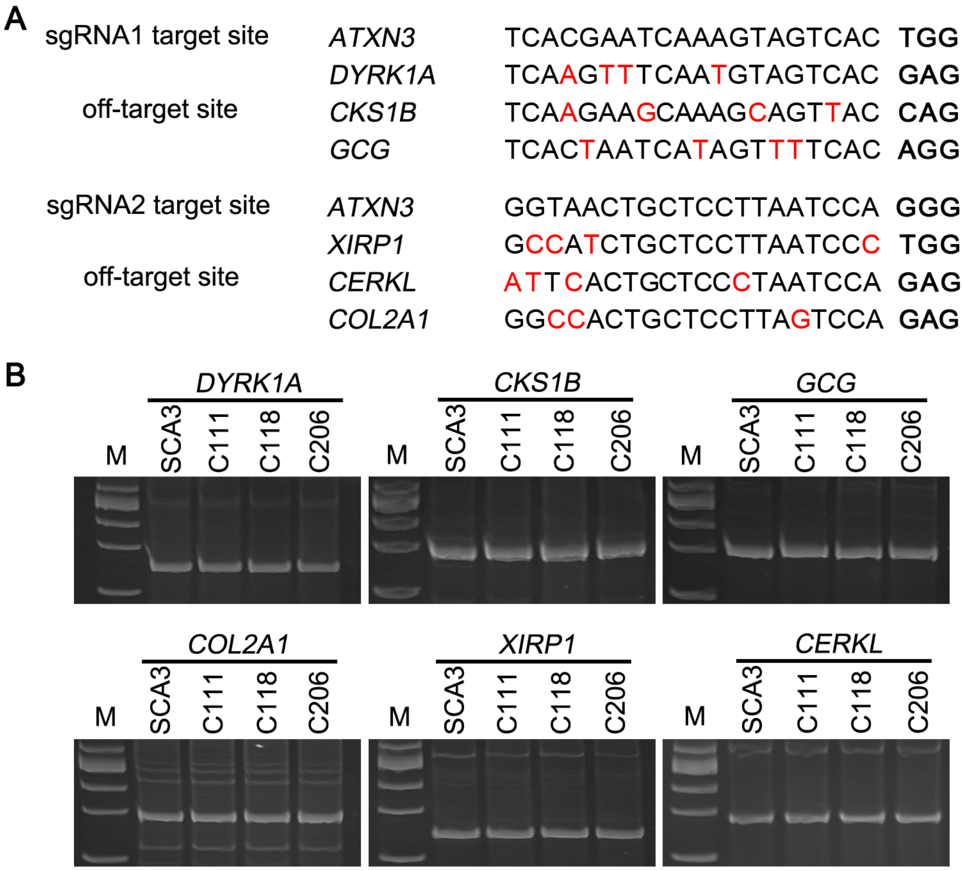

Figure S5. Off-target analysis using polyacrylamide gel electrophoresis. (A) The three most similar potential off-target sites for sgRNA1 and sgRNA2 predicted using the CRISPR design tool developed by Zhang Feng's lab. Red bases indicate sequence differences from the target sequence. (B) No off-target indels were identified at three most similar potential off-target sites in the C111, C118, C206 clones by PAGE.

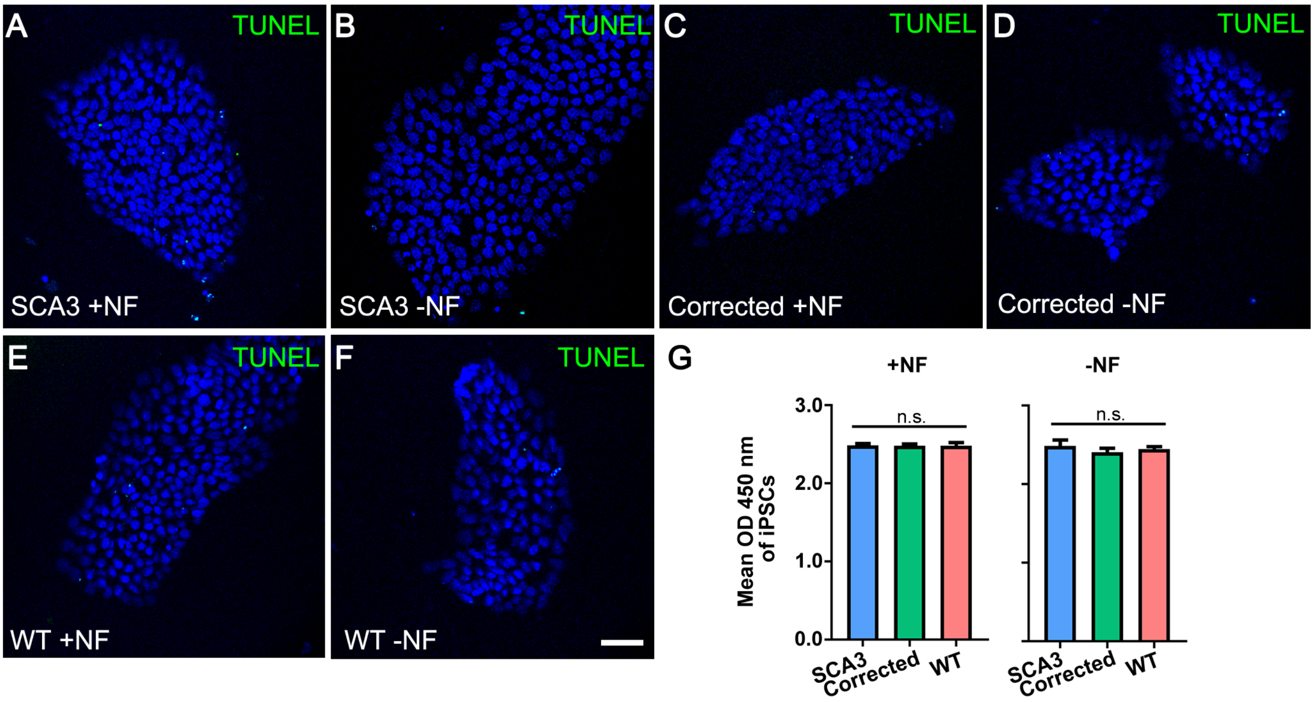

Figure S6. Cell apoptosis and proliferation analysis in SCA3, corrected and WT iPSCs, Related to Figure 4. (A-F) TUNEL staining and percentage of apoptotic cells in SCA3, corrected and WT iPSCs with or without nutritional factors (NF). The scale bars represent 50 μm. (G) Cell proliferation was assayed in SCA3, corrected and WT iPSCs with or without NF using a BrdU cell proliferation assay kit. +NF, normal culture condition supplemented with nutritional factors; -NF, nutritional factor-withdrawal condition. No significant difference. Samples included SCA3 (Pa1 and Pa2), corrected (C-21 and 1A) and WT (WT1 and WT2) iPSCs. Each bar represents mean ± SD with three biological replicates. n.s., not significant.

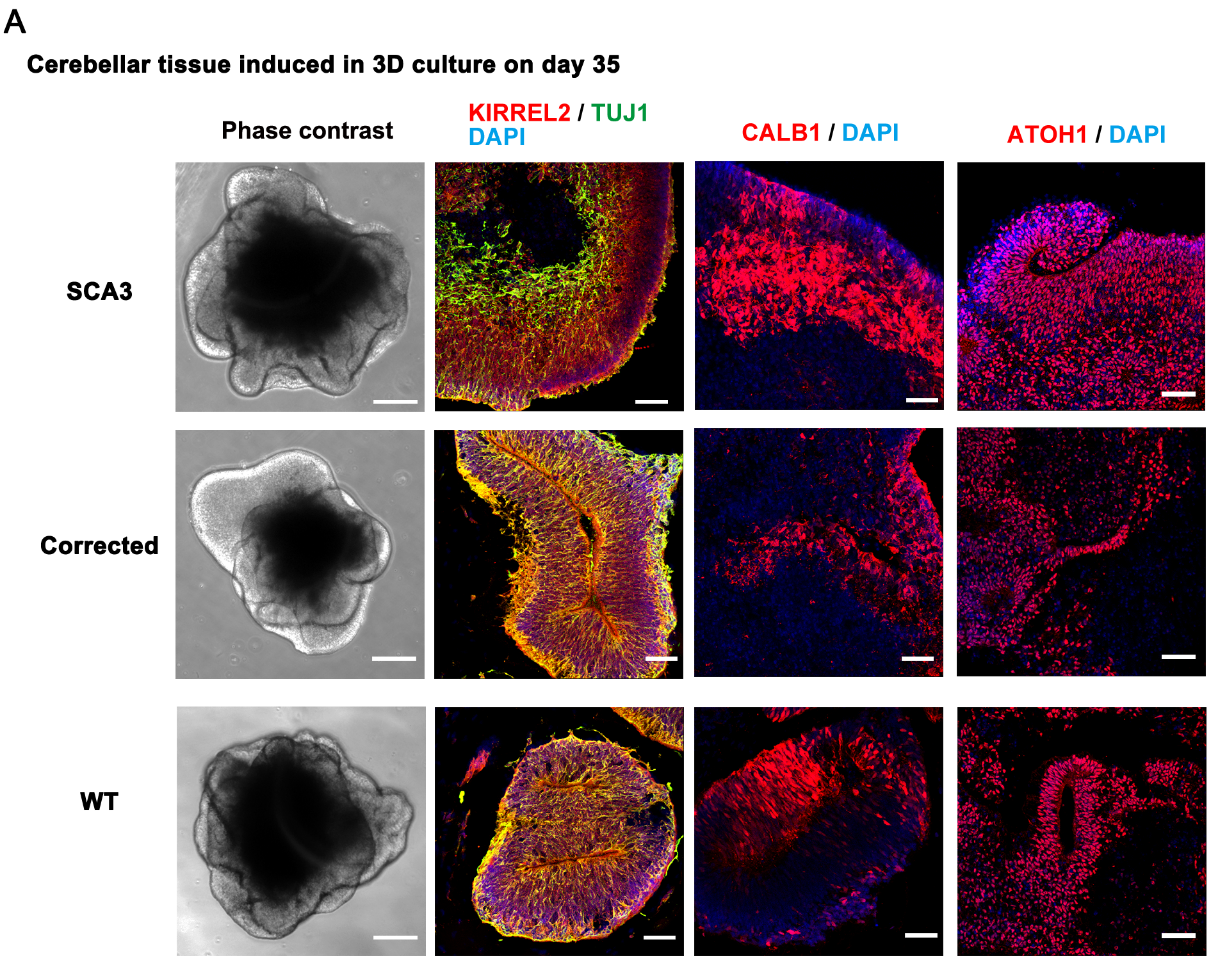
A
Cerebellar tissue induced in 3D culture on day 35
Phase contrast
KIRREL2 / TUJ1
DAPI
CALB1 / DAPI
ATOH1 / DAPI
SCA3
Corrected
WT

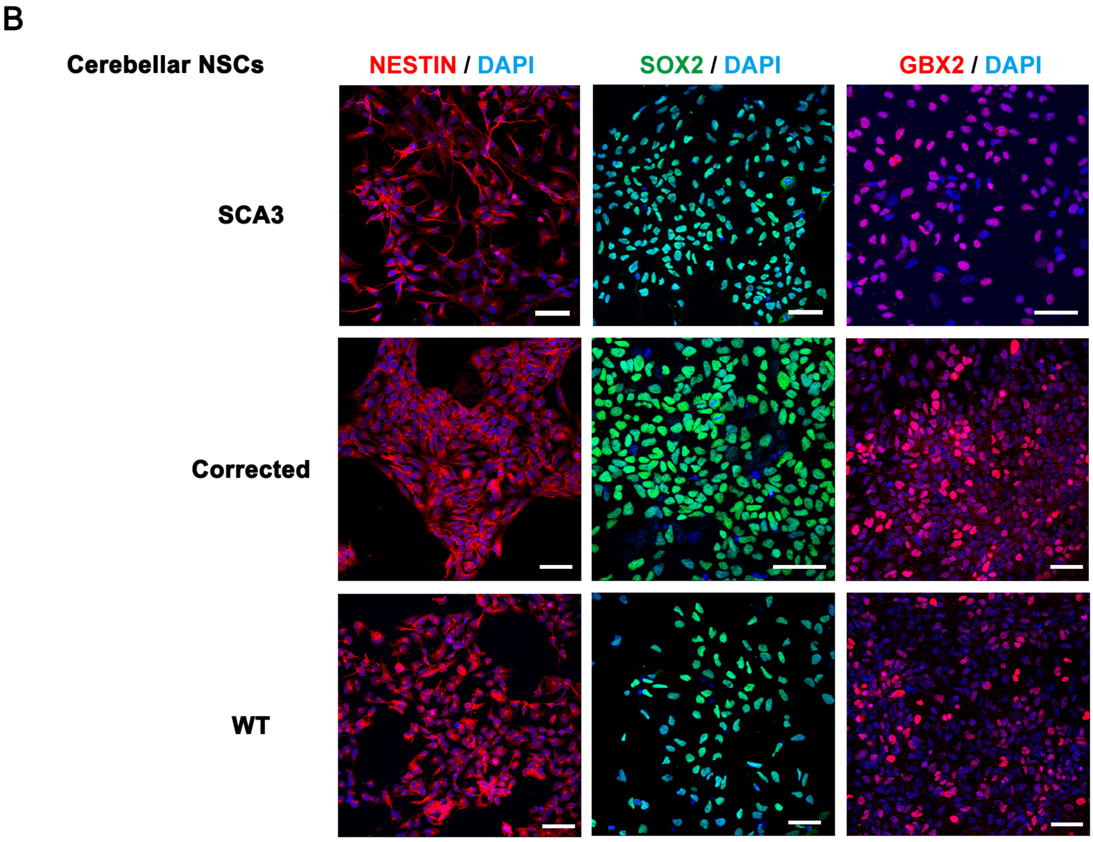
B
Cerebellar NSCs
NESTIN / DAPI
SOX2 / DAPI
GBX2 / DAPI
SCA3
Corrected
WT

Figure S7. Representative immunofluorescence pictures of cerebellar NSCs and 3D-cultured cerebellar tissue, Related to Figure 4. (A) The phase contrast of whole cerebellar tissue and Immunostaining for KIRREL2, TUJ1, CALB1 and ATOH1 in SCA3, corrected and WT 3D-cultured cerebellar tissue on day 35. The scale bars represent 200 μm in phase contrast pictures and 50 μm in immunofluorescence pictures. (B) Expression of cerebellar progenitor-specific markers (GBX2) and neural stem cell markers (SOX2 and NESTIN) in cerebellar NSCs. Samples included SCA3 (Pa1 and Pa2), corrected (C-21 and 1A) and WT (WT1 and WT2) cerebellar NSCs. The scale bars represent 50 μm.

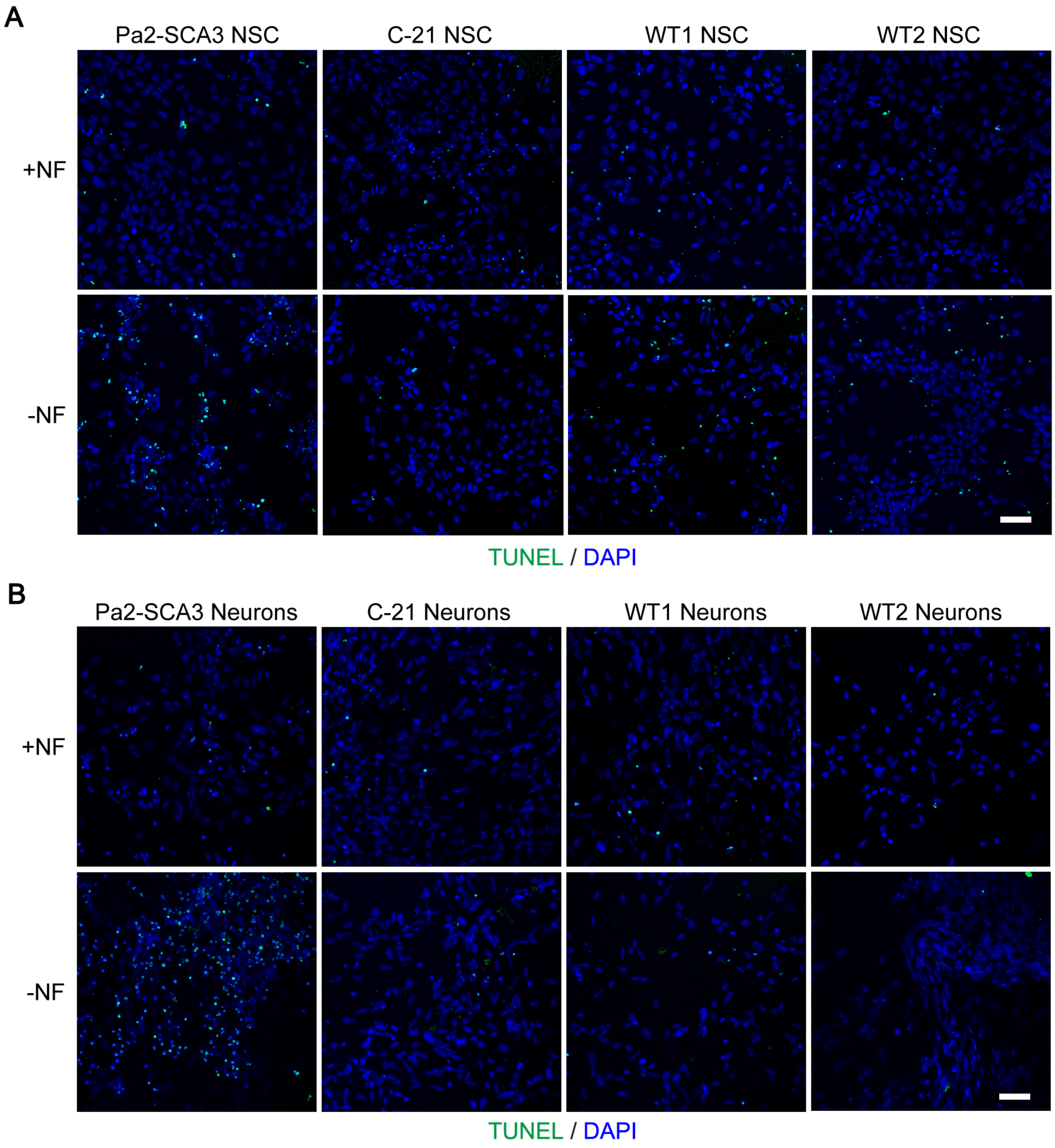

Figure S8. Cell apoptosis analysis in SCA3, corrected and WT cerebellar NSCs and neurons, Related to Figure 4 and 5. (A and B) TUNEL staining and percentage of apoptotic cells in Pa2-SCA3, C-21, WT1 and WT2 cerebellar NSCs (A) and 6-week-differentiated neurons (B) with or without nutritional factors (NF). Experiments were repeated at least three times.

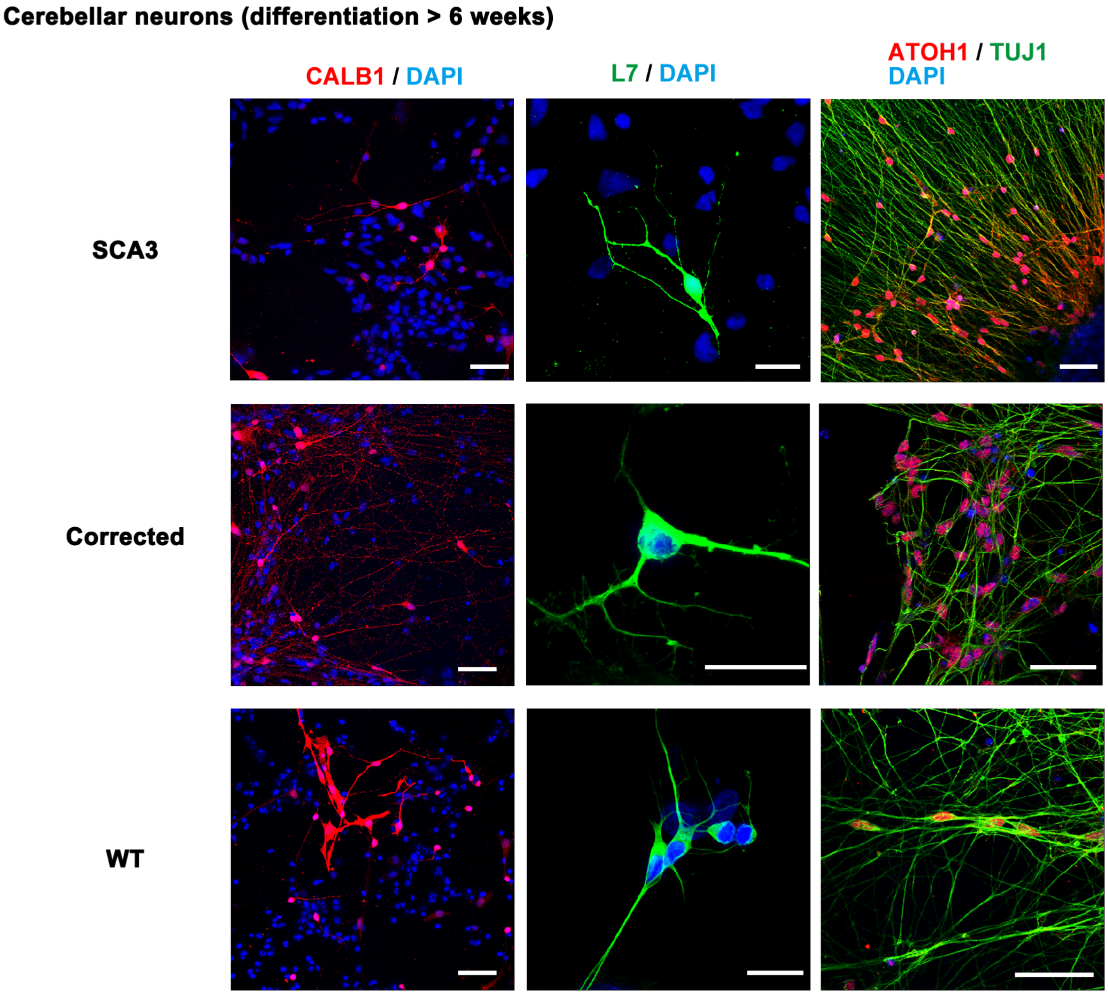

Figure S9. Representative immunofluorescence pictures of cerebellar neurons (differentiation > 6 weeks), Related to Figure 5. Immunostaining for CALB1, L7, ATOH1 and TUJ1 in cerebellar neurons. Samples included SCA3 (Pa1 and Pa2), corrected (C-21 and 1A) and WT (WT1 and WT2) cerebellar neurons. The scale bars represent 20 μm in L7 pictures and 50 μm in other pictures.

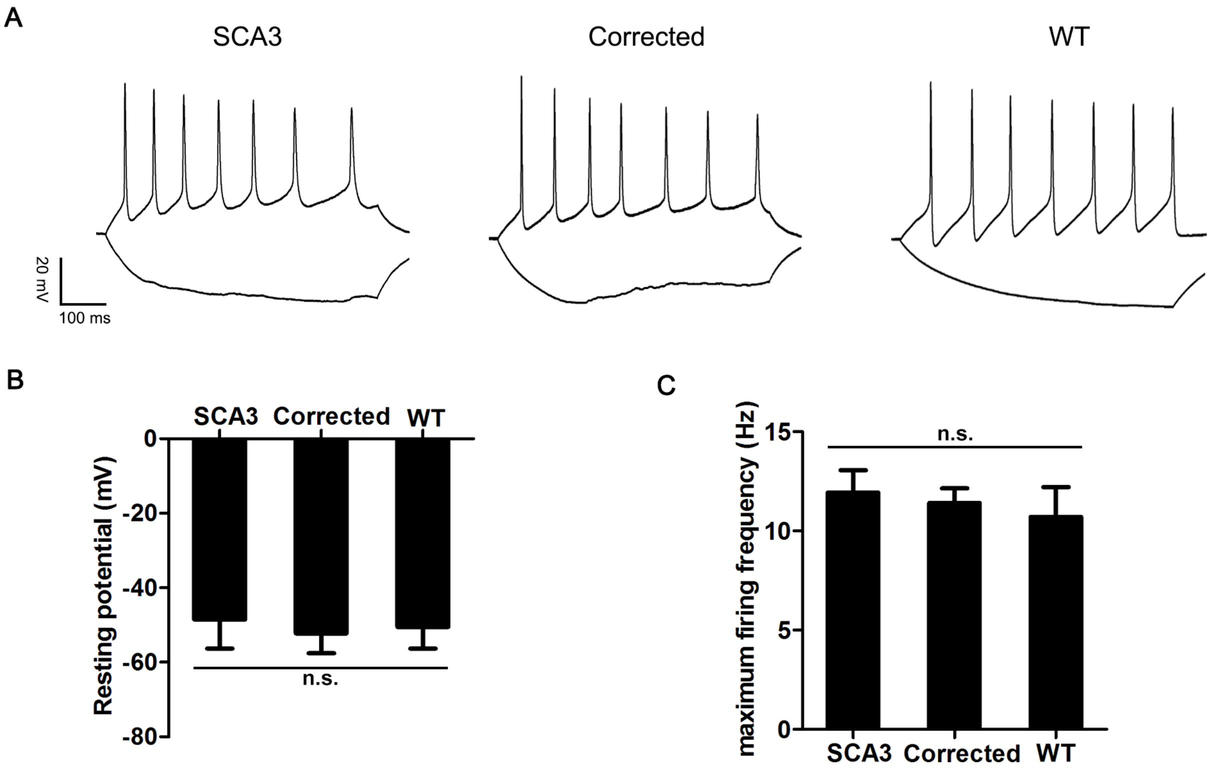

Figure S10. Functional maturation of derived cerebellar neurons in vitro. Whole-cell patch-clamp recorded from cerebellar neurons differentiated for 6 weeks. (A-C) SCA3, corrected and WT cerebellar neurons are able to fire action potentials in response to depolarization (A). There were no significant differences in their resting potential (B) and maximum firing frequency (C). Samples included SCA3 (Pa1 and Pa2), corrected (C-21 and 1A) and WT (WT1 and WT2) neurons. Each bar represents mean ± SD. Experiments were repeated at least three times. n.s., not significant.

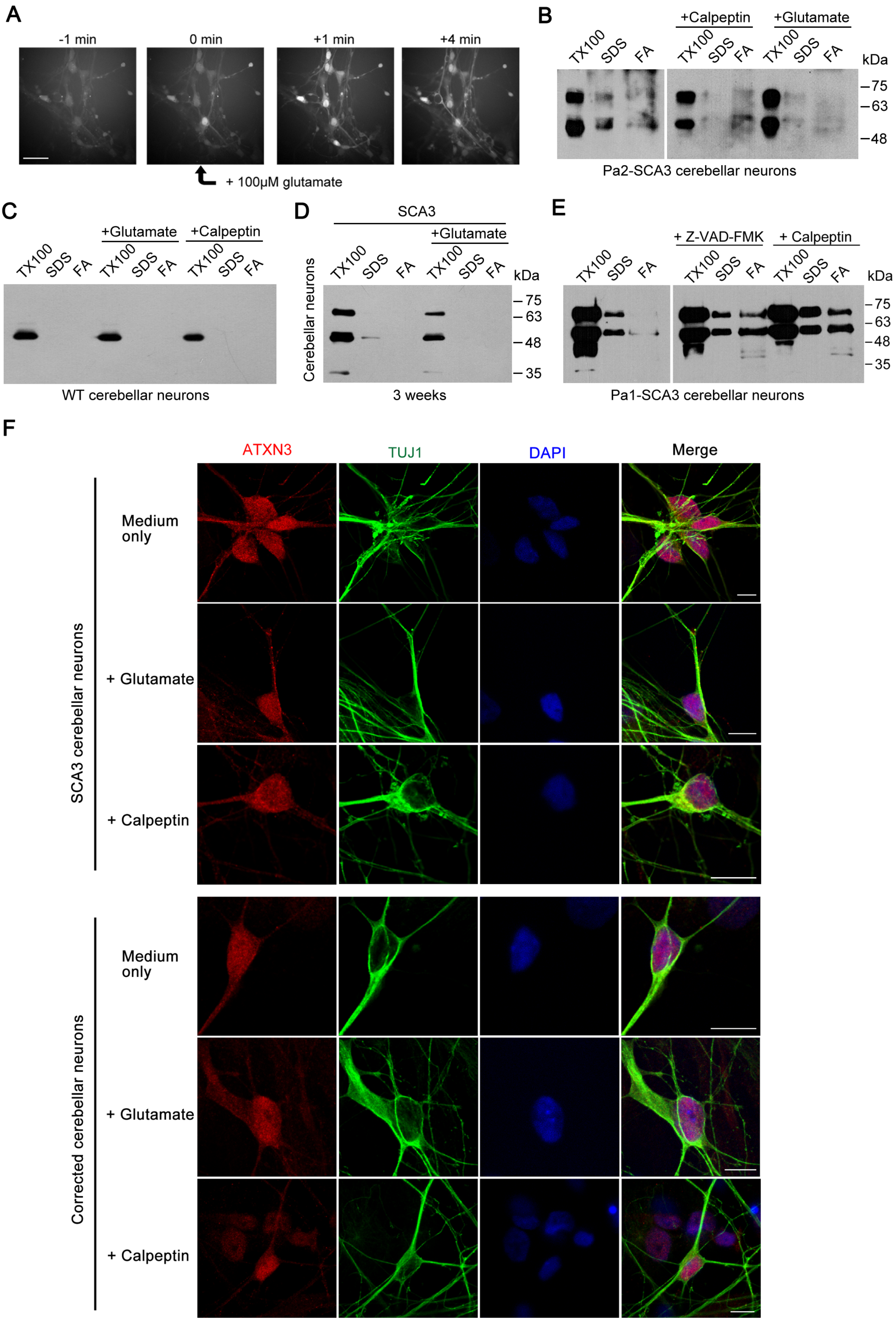
A
-1 min
0 min
+1 min
+4 min
+ 100μM glutamate
B
+Calpeptin
+Glutamate
TX100
SDS
FA
kDa
75
63
48
Pa2-SCA3 cerebellar neurons
C
+Glutamate
+Calpeptin
WT cerebellar neurons
D
SCA3
+Glutamate
Cerebellar neurons
35
3 weeks
E
+ Z-VAD-FMK
+ Calpeptin
Pa1-SCA3 cerebellar neurons
F
ATXN3
TUJ1
DAPI
Merge
SCA3 cerebellar neurons
Medium only
+ Glutamate
+ Calpeptin
Corrected cerebellar neurons

Figure S11. No visible ataxin-3 inclusion bodies or macro-aggregates in SCA3 and corrected cerebellar neurons, Related to Figure 6. (A) Ca^{2+} imaging showed the elevation of the intracellular Ca^{2+} level in neurons after exposure to L-glutamate (100 μM). (B and C) Ataxin-3 aggregation in the presence of the excitatory neurotransmitter L-glutamate (100 μM) or the calpain inhibitor calpeptin (100 μM) in Pa2-SCA3 (B) and WT (C) cerebellar neurons and pan-neuronal cells. (D) SDS-insoluble ataxin-3 aggregates were not detected in 3-week-differentiated SCA3 cerebellar neurons treated with L-glutamate (100 μM). (E) Ataxin-3 aggregation in the presence of the pan-caspase inhibitor Z-VAD-FMK (10 μM) and calpain inhibitor calpeptin (100 μM) in Pa1-SCA3 cerebellar neurons. (F) Immunocytochemistry analysis of ATXN3 in SCA3 and corrected cerebellar neurons (TUJ1) in normal conditions exposed to L-glutamate (100 μM) or calpeptin (100 μM). The scale bars represent 50 μm (A) and 10 μm (F).

Table S2. Analysis of potential off-target sites with whole exome sequencing

Summary of capture statistics

Category	Clone	Total reads	After removing identical reads	Unique (%)	Mapped reads	Mapping (%)
Patients derived clones	Pa1	30,231,866	26,595,903	88%	26,536,414	99.8%
	Pa2	300,196,806	235,784,200	78%	235,454,102	99.9%
Corrected clones	C-21	279,366,532	190,757,027	68%	190,566,263	99.9%
	1A	36,442,158	32,147,294	88%	32,072,738	99.8%

Identification of potential off-target sites in two corrected clones

Clone	sgRNA	Sequence	Target sites	Potential off-target sites in exons	Mutations in off-target sites and flanking100 bp
C-21	sgRNA1	TCACGAATCAAAGTAGTCACTGG	1	3	0
	sgRNA2	GGTAACTGCTCCTTAATCCAGGG	1	11	0
1A	sgRNA3	CTTATGAATAGTTTTTCTCATGG	1	25	0
	sgRNA4	GATGTGAACTCTGTCCTGATAGG	1	31	0

Off-target analysis based on whole exome sequencing (WES). Potential off-target sites for each sgRNA predicted using Zhang laboratory CRISPR design tool (Table S1). WES was performed on the two corrected iPSC clones (C-21 and 1A) and two SCA3 iPSC clones (Pa1 and Pa2-SCA3).

Supplementary Materials and Methods

Ethical statement

SCA3 patients and healthy individuals in this manuscript have signed a written informed consent for donating isolated epithelial cells from urinary samples for stem cell research. The human subject protocol was reviewed and approved by the institutional committee for human ethics at Institute of Biophysics, Chinese Academy of Sciences. All procedures concerning the care and use of animals were performed according to IACUC approved protocols.

Donors and sgRNA expression vector construction

For donor vector construction, the DNA was synthesized and ligated into a pUC18 cloning vector (Biomed). We used two donors for genetic editing, the Donor-608 and Donor-2201. The letters represent targeting sequences in each donor plasmid. Green shaded letters represent CAG repeat sequence in donor plasmid. Red letters represent the Cas9 protein blocking mutations (CBMs) made in the donor arms.

Donor-608 (608bp, 13 CAG repeats)

5'-TCAGACTAACTGCTCTTGCATTCTTTTAATACCAGTGACTACTTTGATTCGTGAAACAATGTATTTTCCTTATGA
ATAGTTT**C**TC**C**CATGGTGTATTTATTCTTTTAAGTTTTGTTTTTTAAATATACTTCACTTTTGAATGTTTCAGACAGC
AGCAAAAGCAGCAACAGCAGCAGCAGCAGCAGCAGCAGGGGGA**T**CTATCAGGACAGAGTTCACATCCATGTGA
AAGGCCAGCCACCAGTTCAGGAGCACTTGGGAGTGATCTAGGTAAGGCCTGCTCACCATTCATCATGTTCGCTA
CCTTCACACTTTATCTGACATACGAGCTCCATGTGATTTTTGCTTTACATTATTCTTCATTCCCTCTTTAATCATAT
TAAGAATCTTAAGTAAATTTGTAATCTACTAAATTTCCCTGGATTAAGGAGCAGTTACCAAAAGAAAAAAAAAAAA
AAAAGCTAGATGTGGTGGCTCACATCTGTAATCCCAGCACTTTGGGAAACCAAGGCAGGAGAGGATTGCTAGAA
CATTTAATGAATACTTTAACATAATAATTTAAACTTCACAGTAATTTGTACAGTCTCCAAAAATTCCTTAGACATCA
TGGA-3'

Donor-2201 (2201bp, 13 CAG repeats)

5'-TCTTTTTGAAAAAATTACAGAGGAGCTTAATGCAATCAGTATTACTTAAAATCTGATAATGTGTGTTAAATAGTA
GTTTTCATTTATTTCATTTATCAGGTGTTCAGTGAATGCTTACTATGTAACAGCACAGTTATCAGCACTGGGGAAA
TAGATGAGTAAGATAAGATTTGCACTTTCATTAGCTTACATGCCATAAAGAGGGAAATAAAGAGAACACCAGATG
ATGATAAGTTTATGCTGAGAATTAAAATGAAGTGATGAAATAATGGGAATGTCAGGTGGCTACTTTTGGTGGGAT
GGTCAGGAAAGGCATCTCTGGGGAGATAAATTTTAAGCTCAGACCTGAGTGAAAAGAATGAGCCAGCCATGGAA
ACATTATGTTAACTCACATGGTAGTTTGAAATGCTTTATCTGATCAAAGGTACTTATTTTTGGTGACTTTCAACAAT
ATTAAGGGTCTATAAACCAACACTCATTTGCATAAGAATAACTACCAGTGAATCTTTTTGTATGATAGGTTTTTTGT
TTGTTGTTTTTTTGAGACAGAGTCTCGCTCTGTCGCCCAGGCTGGAGTGCAGTGGCGCGATCTTGGCTCACTGC
AACCTCTACCTCCCCGGTTCAAGTGATTCTCCTGCCTCAGCCTCCCAAAGTAGCTGGGATTACAGGTGCCTGCC
ACCACGCCTGGCTAATTTTTGTATTTTTAGTAGAGATGGGGTTTCACCGTGTTGTCCAGGCTCGTGTCAAACTTC
TGACCTCAAGCCATCCACCCGCCTCGGCCTCCCAAAGTGCTGGGATTACAGGTGTGAGCCACCACTCCTGGCC
ATGATAGGTTATTTTGTGATGAAAATACCTACCTCTTAATTTGTCTGATAAATTTAAATTTTATGTCTAGATTTCCTA
AGATCAGCACTTCCATATTTTAAAGTAATCTGTATCAGACTAACTGCTCTTGCATTCTTTTAATA**GG**AGTGACTAC

TTTGATTCGTGAAACAATGTATTTTCCTTATGAATAGTTTTTCTCATGGTGTATTTATTCTTTTAAGTTTTGTTTTTT
AAATATACTTCACTTTTGAATGTTTCAGACAGCAGCAAAAGCAGCAACAGCAGCAGCAGCAGCAGCAGCAGGGG
GACCTATCAGGACAGAGTTCACATCCATGTGAAAGGCCAGCCACCAGTTCAGGAGCACTTGGGAGTGATCTAG
GTAAGGCCTGCTCACCATTCATCATGTTCGCTACCTTCACACTTTATCTGACATACGAGCTCCATGTGATTTTTG
CTTTACATTATTCTTCATTCCCTCTTTAATCATATTAAGAATCTTAAGTAAATTTGTAATCTACTAAATTTGGCTGGA
TTAAGGAGCAGTTACCAAAAGAAAAAAAAAAAAAAAAGCTAGATGTGGTGGCTCACATCTGTAATCCCAGCACTT
TGGGAAACCAAGGCAGGAGAGGATTGCTAGAACATTTAATGAATACTTTAACATAATAATTTAAACTTCACAGTAA
TTTGTACAGTCTCCAAAAATTCCTTAGACATCATGGATATTTTTCTTTTTTTGAGATGGAGTCTTGCTCTGTCACC
CAGGCTGGAGTGCAGTGTCGCGATCTCGGCTCACTGCAAGCTCTGCTTCCTGGGTTCATGGCATTCTCCTGCC
TCAGCCTCCTGAGTAGCTGGGACTACAGGCGCCCGCCACATCGCCTGGCTAATTTTTTGTATTTTTAGTAGAGA
CAGGGTTTCACCATGTTAGCCAGGATGGTCTCAATCTCCTGACCTCATGATCCGCCCGCCTCGGCCTCCCAAAG
TGCTGGGATTACAGGCGTGAGCCATCACGTCCGGCCAGAAATCATGAATATTAGTAGGTGAAAAATAAACACAT
TTTACCACCTGGAAAATGAAAAATACTTGAGTATAATCTAAATAACAATGGGAAGTGCAGAGTTACTTTCCAGGT
CTCGGTTTAAATATGTCTTAAACTTTGGCCAATTAGTAGTAGAAGTTGAGAGAAAAAGTAACTATCTGACAAAGAA
ATTATAAGCAGAATATATAAAGAACTCTTAAAACTGAATAATCAGAAAACAACTCAATAAAAAGGTGAAGGATTTG
AAAAGATATTTCACCAAATAAGACATAGGGATGACAAATAAGCACATGAAAAGACTCTCAGCATCACTAGTCACA
GGGAAATGCACGATAAAACC-3’

Construction of sgRNA expression vector was performed as previously described (Song et al., 2022; Song et al., 2019). Briefly, the annealed oligonucleotides for each sgRNA were cloned into a pGL3-sgRNA expression vector driven under U6 promoter for human cell transfection.

Oligo location	Oligo sequence
sgRNA1 top strand	5’-CCGGTCACGAATCAAAGTAGTCAC-3’
sgRNA1 bottom strand	5’-AAACGTGACTACTTTGATTCGTGA-3’
sgRNA2 top strand	5’-CCGGGGTAACTGCTCCTTAATCCA-3’
sgRNA2 bottom strand	5’-AAACTGGATTAAGGAGCAGTTACC-3’
sgRNA3 top strand	5’-CCGGCTTATGAATAGTTTTTCTCA-3’
sgRNA3 bottom strand	5’-AAACTGAGAAAAACTATTCATAAG-3’
sgRNA4 top strand	5’-CCGGGATGTGAACTCTGTCCTGAT-3’
sgRNA4 bottom strand	5’-AAACATCAGGACAGAGTTCACATC-3’

Red letters represent sgRNA sequences corresponding to gene locus. The annealed oligos were cloned between two BsaI restriction sites downstream of the U6 promoter in the sgRNA expression vector for use in human cells.

SCA3 iPSCs transfection and screening

One day before transfection, at least 3 × 10^6 iPS cells/well were planted on Geltrex-coated 6-well plate in Essential 8 medium supplemented with 10μM ROCK inhibitor Y-27632 (Sigma). For genetic correction in SCA3 iPSCs, cells were transfected with 1 μg of each sgRNA plasmid, 2 μg of donor DNA plasmid, and 1 μg of pCas9-GFP plasmid (Addgene number: 44719) by Lipofectamine®

LTX reagent (Invitrogen) according to the manufacturer's protocol. After 1 day, iPS cells were sorted by FACS (FACSAriaIII, BD) and 3000 cells/well cultured on feeder-coated 6-well plate in iPSC medium supplemented with Y-27632 (first 2 day) for another 2 weeks until single clones could be picked for genotyping. Half of the single clones were placed into a 0.2-ml tube for genomic DNA extraction, and the other half was placed into a 96-well plate for maintenance. Genomic DNA from individual iPS cell clones were quickly extracted by DNA extraction solution 1.0 (BuccalAmp). PCR and 3% agarose gel electrophoresis were used to detect genomic modification. The expected PCR band was purified for further sequencing analysis.

**Primers for genotyping**	
**Primer name**	**Sequence (5' to 3')**
P1-f	TGGGGATTTGTATTACATTGTTTC
P1-r	GAATGGTGAGCAGGCCTTAC
P2-f	TTTCCTAAGATCAGCACTTCCA
P2-r	GTGCCATGGTGTCTCACTGT
P3-f	TTTCCTAAgATCAGCACTTCCA
P3-r	ACATGGAGCTCGTATGTCAGA
P4-f	CCAGTGACTACTTTGATTCGTGA
P4-r	AAGCAGAGCTTGCAGTGAGC
D608-T-f	GTAAAACGACGGCCAGT
D608-T-r	ACCAGTGACTACTTTGATTCGTGA
D2201-T-f	GTAAAACGACGGCCAGT
D2201-T-r	CACTCCTGGCCATGATAGGT

ATXN3 cDNA sequencing

Total RNAs were purified from iPSCs using Trizol according to the manufacture's protocol (Invitrogen). A total of 1 μg of RNA was converted to cDNA by StarScript II First-strand cDNA Synthesis Kit (GenStar). PCR and 3% agarose gel electrophoresis were used to detect *ATXN3* mRNA for subsequent sequencing analysis. The primers are: *ATXN3* forward 5'-CGCAGGGCTATTCAGCTAAG-3'; *ATXN3* reverse 5'- AGTCCTACAACCGACGCATT-3'.

Characterization of iPSCs

Immunocytochemistry analysis of iPSCs was performed with human pluripotency markers antibody including OCT4 (Proteintech), SSEA4 (Signaling Technology), NANOG (Proteintech), SOX2 (Proteintech). DAPI was used for nuclear visualization. The images were captured by Leica

TSC SPE microscope. Karyotyping analyses were performed as described previously (Xue et al., 2013).

For embryoid bodies (EBs) formation assay, human iPSC clones were detached and plated on petri dishes in iPSC medium (without bFGF) to from EBs. Medium was changed every other day. At day 8, EBs were plated on gelatin-coated (Sigma) tissue culture coverslips. After another 8 days culture, cells were stained with markers from three germ layers: AFP (endoderm), VEMINTIN (mesoderm), and TUJ1 (ectoderm).For analysis in the teratoma assay, cells from a confluent 60-mm dish were harvested by accutase (Millipore) digestion, resuspended in iPSC medium supplemented with Y-27632, and injected into immunodeficient mice. Eight weeks later, subcutaneous teratoma was isolated and fixed in 4% paraformaldehyde. Paraffin sections were prepared and stained with hematoxylin/eosin.

Cerebellar NSCs and pNSCs derivation from iPSCs

To generate cerebellar NSCs, we first performed neural differentiation to cerebellar-plate-like neuroepithelium from iPSCs using serum-free suspension culture previously described (Muguruma et al., 2015) with slight modifications. Briefly, on day 0, human iPSCs were dissociated to single cells and reaggregated to EBs using low-binding 96-well plate (Corning) in differentiation medium (8000 cells per well) with 50 μM Y-27632 (Sigma) only for the first 4 days. The components of differentiation medium and the period of culture were described previously (Muguruma et al., 2015). EBs were fed every other day and cultured until day 21 to transform to 90-mm Petri dish in suspension culture during days 21-35 using Neurobasal medium (Invitrogen) supplemented with N2 supplement (1:100, Invitrogen), L-glutamine and penicillin-streptomycin (both from Millipore).

Next, we purified a rosette-type, self-renewing CPNE stem cells population from cerebellar neuroepithelium cultured for about 35 days according to previous protocol (Koch et al., 2009; Tailor et al., 2013). Neural tube-like structures developed in the EBs outgrowth expressed CPNE-specific marker KIRREL2 (Figure 4E). These structures were mechanically separated with needle and dissociated to single cells with 0.25% Trypsin/EDTA (Millipore). Cells were plated on poly-ornithine/laminin (both from Sigma) coated dishes in NSCs medium containing DMEM/F12 medium supplemented with N2 supplement (1:100), B27 supplement (1:1000, Invitrogen), 10 ng/ml EGF (Invitrogen), 10 ng/ml bFGF (R&D), 2 mM L-glutamine, 20 μg/ml insulin (Sigma), penicillin-streptomycin. Cells were passaged at a ratio of 1:2–1:3 every 2–3 days using trypsin.

For pNSCs derivation, we performed a rapid neural induction using PSC Neural Induction Medium (Invitrogen) as a manufacture's protocol. Briefly, iPSCs were cultured in PSC Neural Induction Medium containing of Neurobasal medium supplemented with Neural Induction Supplement on Geltrex. After 7 days of neural induction, NSCs are ready to be harvested and expanded with Neural Expansion Medium consisting of 49% Neurobasal medium, 49% DMEM/F12 medium, and 2% Neural Induction Supplement.

Off-Target Analysis

First, we identified potential off-target sites for each sgRNA using the CRISPR design tool (http://crispr.mit.edu) with the synthetic 20-nt sgRNA sequence plus 5'-NGG "proto-spacer adjacent motif" (PAM) sequence (Table S1). Next, off-target analysis was determined by WES and PAGE. For WES analysis, the off-target was detected by whether the variants were located at potential off-target site. PAGE was performed on detection of the three most similar off-target sites for sgRNA1 and sgRNA2. The selected off-target sites were amplified with PCR reaction followed with standard PAGE assay as described (Zhu et al., 2014).

**Primers for off-target analysis**	
**Primer name**	**Sequence (5' to 3')**
CKS1B-f	GCAAGCTACTTTTCAGCCTCA
CKS1B-r	ACTCATCTGGCTGGGCTATG
DYRK1A-f	GAGAAGAGTCCCCCATGACA
DYRK1A-r	TGATTCAAGCAGCTTTGCAC
GCG-f	GCAACGTTCCCTTCAAGACA
GCG-r	CACTCCAACAAAGTCTGCTTTT
CERKL-f	TTTCAGGATTTGAGCTCATCAAT
CERKL-r	GACAGGGATGGGTTATTCTTTTT
COL2A1-f	ACTGGCAAGCAAGGAGACAG
COL2A1-r	GCACCCTGAGGAGAGAGTGA
XIRP1-f	GTGAATTTGCACACCACTGC
XIRP1-r	AGACCAACGCTGTGTCCAGT

Whole Exome Sequencing

The exome sequences were efficiently enriched from 1 μg genomic DNA using Agilent liquid capture system (Agilent SureSelect Human All Exon V6) according to the manufacturer's protocol. DNA library were sequenced on Illumina Hiseq 4000 for paired-end 150bp reads. The sequencing

depth was 50X. Valid sequencing data is mapped to the reference genome (UCSC hg19) by Burrows-Wheeler Aligner (BWA) software (Li and Durbin, 2009) to obtain the original mapping result in BAM format. Subsequently, other bioinformatics tools were used for analysis of samples, including Samtools (Li et al., 2009), Picard, CoNIFER, PolyPhen-2, SIFT, MutationTaster and CADD. The variants including SNVs and indels were determined whether they were within the potential off-target sites ± 100 flanking base pairs.

Electrophysiological analysis

The Whole-cell patch-clamp recordings were performed as previously described (Koch et al., 2011). Briefly, cells were cultured on coverslips for 6 weeks in differentiation medium and then were performed for electrophysiological analysis. All recordings were performed at room temperature with the extracellular solution containing (in mM): 150 NaCl, 5 KCl, 2 $CaCl_2$, 1 $MgCl_2$, 10 HEPES and 15 D-glucose (pH 7.2). The patch pipette solution contained the following (in mM): 140 K-gluconate, 5 KCl, 0.2 EGTA, 2 $MgCl_2$ and 10 HEPES (pH 7.2). Electrode resistance ranged from 5 to 7 MΩ. The whole cell recording was conducted with HEKA EPC-10 patch-clamp amplifier with associated software (PatchMaster, HEKA Electronic Inc., Germany). The currents were typically digitized at 10 KHz. Macroscopic records were filtered at 2.9 KHz. All electrophysiological recordings were analyzed with Clampfit software (version 10.0; Axon Instruments).

Statistical Analysis

Statistical tests were performed with the software GraphPad Prism 5. Statistical significance was tested with unpaired t test for two-group comparisons or one-way ANOVA test for multi-group comparison.

Supplemental references

Koch, P., Breuer, P., Peitz, M., Jungverdorben, J., Kesavan, J., Poppe, D., Doerr, J., Ladewig, J., Mertens, J., Tuting, T., *et al.* (2011). Excitation-induced ataxin-3 aggregation in neurons from patients with Machado-Joseph disease. Nature *480*, 543-546.

Koch, P., Opitz, T., Steinbeck, J.A., Ladewig, J., and Brustle, O. (2009). A rosette-type, self-renewing human ES cell-derived neural stem cell with potential for in vitro instruction and synaptic integration. Proceedings of the National Academy of Sciences of the United States of America *106*, 3225-3230.

Li, H., and Durbin, R. (2009). Fast and accurate short read alignment with Burrows-Wheeler transform. Bioinformatics *25*, 1754-1760.

Li, H., Handsaker, B., Wysoker, A., Fennell, T., Ruan, J., Homer, N., Marth, G., Abecasis, G., Durbin, R., and Genome Project Data Processing, S. (2009). The Sequence Alignment/Map format and SAMtools. Bioinformatics *25*, 2078-2079.

Muguruma, K., Nishiyama, A., Kawakami, H., Hashimoto, K., and Sasai, Y. (2015). Self-organization of polarized cerebellar tissue in 3D culture of human pluripotent stem cells. Cell Rep *10*, 537-550.

Song, G., Zhang, F., Tian, C., Gao, X., Zhu, X., Fan, D., and Tian, Y. (2022). Discovery of potent and versatile CRISPR-Cas9 inhibitors engineered for chemically controllable genome editing. Nucleic acids research *50*, 2836-2853.

Song, G., Zhang, F., Zhang, X., Gao, X., Zhu, X., Fan, D., and Tian, Y. (2019). AcrIIA5 Inhibits a Broad Range of Cas9 Orthologs by Preventing DNA Target Cleavage. Cell Rep *29*, 2579-2589 e2574.

Tailor, J., Kittappa, R., Leto, K., Gates, M., Borel, M., Paulsen, O., Spitzer, S., Karadottir, R.T., Rossi, F., Falk, A., *et al.* (2013). Stem cells expanded from the human embryonic hindbrain stably retain regional specification and high neurogenic potency. The Journal of neuroscience : the official journal of the Society for Neuroscience *33*, 12407-12422.

Xue, Y., Cai, X., Wang, L., Liao, B., Zhang, H., Shan, Y., Chen, Q., Zhou, T., Li, X., Hou, J., *et al.* (2013). Generating a non-integrating human induced pluripotent stem cell bank from urine-derived cells. PloS one *8*, e70573.

Zhu, X., Xu, Y., Yu, S., Lu, L., Ding, M., Cheng, J., Song, G., Gao, X., Yao, L., Fan, D., *et al.* (2014). An efficient genotyping method for genome-modified animals and human cells generated with CRISPR/Cas9 system. Scientific reports *4*, 6420.
